# Nesprin-1α-Dependent Microtubule Nucleation from the Nuclear Envelope via Akap450 Is Necessary for Nuclear Positioning in Muscle Cells

**DOI:** 10.1016/j.cub.2017.08.031

**Published:** 2017-10-09

**Authors:** Petra Gimpel, Yin Loon Lee, Radoslaw M. Sobota, Alessandra Calvi, Victoria Koullourou, Rutti Patel, Kamel Mamchaoui, François Nédélec, Sue Shackleton, Jan Schmoranzer, Brian Burke, Bruno Cadot, Edgar R. Gomes

**Affiliations:** 1 Sorbonne Universités UPMC Université Paris 06, INSERM U974, CNRS FRE3617, Center for Research in Myology, GH Pitié Salpêtrière, 47 Boulevard de l’Hôpital, 75013 Paris, France; 2 Institute of Medical Biology, Agency for Science, Technology and Research (A^∗^STAR), 8A Biomedical Grove, No. 06-06 Immunos, Singapore 138648, Singapore; 3 Institute of Molecular and Cell Biology, Agency for Science, Technology and Research (A^∗^STAR), 61 Biopolis Drive, No. 07-48A Proteos, Singapore 138673, Singapore; 4 Cell Biology and Biophysics Unit, European Molecular Biology Laboratory, 69117 Heidelberg, Germany; 5 Department of Molecular and Cell Biology, Henry Wellcome Building, University of Leicester, Lancaster Road, Leicester LE1 7RH, UK; 6 Charité-Universitätsmedizin Berlin, Virchowweg 6, 10117 Berlin, Germany; 7 Instituto de Medicina Molecular, Faculdade de Medicina, Universidade de Lisboa, Avenida Professor Egas Moniz, 1649-028 Lisbon, Portugal

**Keywords:** nuclear envelope, nuclear positioning, centrosome, Nesprin-1, Nesprin-1α, Akap450, skeletal muscle, cytosim computer simulation, microtubules, non-centrosomal MTOC

## Abstract

The nucleus is the main microtubule-organizing center (MTOC) in muscle cells due to the accumulation of centrosomal proteins and microtubule (MT) nucleation activity at the nuclear envelope (NE) [1, 2, 3, 4]. The relocalization of centrosomal proteins, including Pericentrin, Pcm1, and γ-tubulin, depends on Nesprin-1, an outer nuclear membrane (ONM) protein that connects the nucleus to the cytoskeleton via its N-terminal region [5, 6, 7]. Nesprins are also involved in the recruitment of kinesin to the NE and play a role in nuclear positioning in skeletal muscle cells [8, 9, 10, 11, 12]. However, a function for MT nucleation from the NE in nuclear positioning has not been established. Using the proximity-dependent biotin identification (BioID) method [13, 14], we found several centrosomal proteins, including Akap450, Pcm1, and Pericentrin, whose association with Nesprin-1α is increased in differentiated myotubes. We show that Nesprin-1α recruits Akap450 to the NE independently of kinesin and that Akap450, but not other centrosomal proteins, is required for MT nucleation from the NE. Furthermore, we demonstrate that this mechanism is disrupted in congenital muscular dystrophy patient myotubes carrying a nonsense mutation within the *SYNE1* gene (*2*3560 G>T) encoding Nesprin-1 [15, 16]. Finally, using computer simulation and cell culture systems, we provide evidence for a role of MT nucleation from the NE on nuclear spreading in myotubes. Our data thus reveal a novel function for Nesprin-1α/Nesprin-1 in nuclear positioning through recruitment of Akap450-mediated MT nucleation activity to the NE.

## Highlights

• BioID of Nesprin-1α identifies centrosomal proteins at myotube nuclear envelope
• Nesprin-1α-containing LINC complexes recruit Akap450 to myotube nuclear envelope
• Akap450 is required for microtubule nucleation at the nuclear envelope
• Microtubule nucleation at the nuclear envelope is involved in nuclear positioning

## Graphical Abstract

**Figure:**
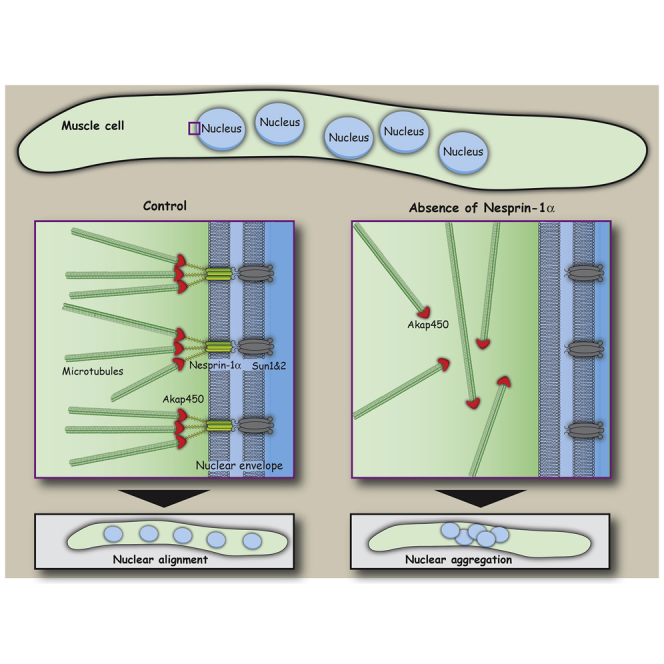

## Teaser

Gimpel et al. find that LINC complexes comprising Nesprin-1α and Sun1/2 are required for the recruitment of centrosomal proteins and Akap450-mediated microtubule nucleation activity to the myotube nuclear envelope. Absence of microtubule nucleation from the nucleus affects nuclear positioning in muscle cells *in vitro* and in computer simulations.

## Results and Discussion

### BioID Screen Identifies Nesprin-1α-Associated Centrosomal Proteins in Myotubes

**Figure 1:**
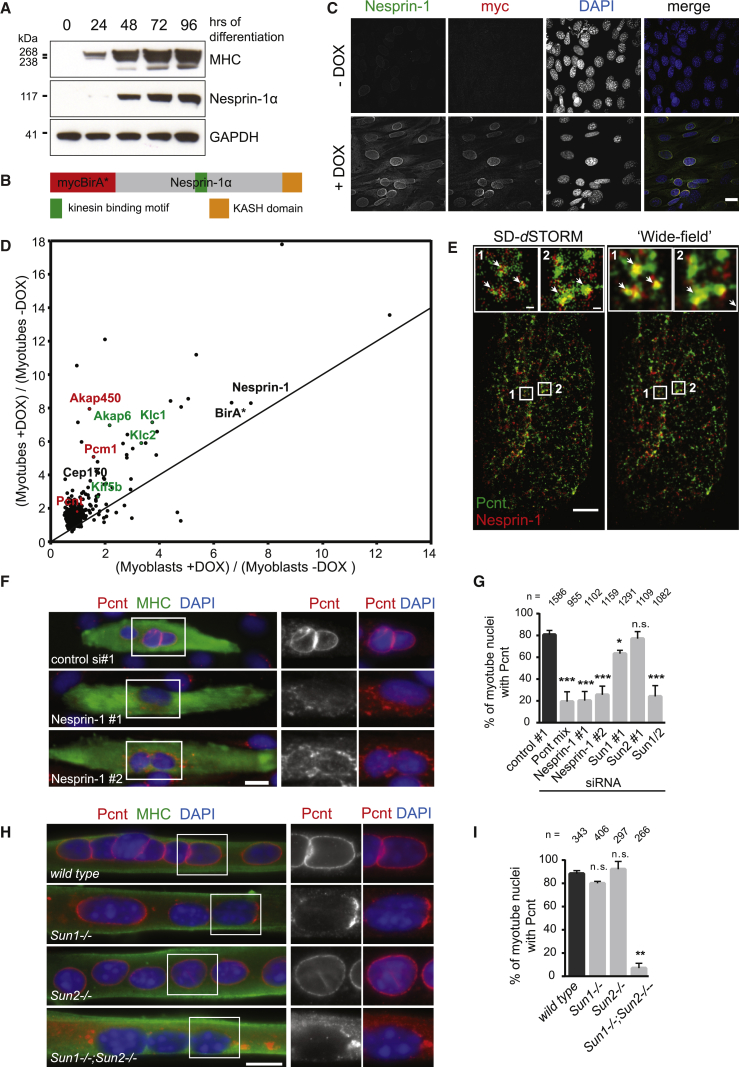
LINC Complex Comprising Nesprin-1 and Sun1/Sun2 Is Required for NE Localization of Centrosomal Proteins during Myogenic Differentiation (A) C2C12 myoblasts were differentiated by serum starvation for the indicated time points (hours of differentiation). Cell lysates were analyzed by western blot using antibodies against myosin heavy chain (MHC), Nesprin-1α (MANNES1E monoclonal antibody [mAb]), and GAPDH. (B) Schematic of Nesprin-1α fused to myc-BirA^∗^ (BioID-Nesprin-1α) for BioID. (C) Non-differentiated C2C12 cells stably expressing doxycycline-inducible myc-BirA^∗^-Nesprin-1α were treated with (+DOX) or without (−DOX) doxycycline, fixed and stained for Nesprin-1 (green, clone 9F10), myc (red), or nuclei (DAPI, blue). The scale bar represents 20 μm. (D) Depicted are normalized quantities of proteins purified on streptavidin beads in BioID-Nesprin-1ɑ-expressing C2C12 myotubes (y axis) and myoblasts (x axis) treated with biotin. Each protein quantity is the ratio of the amount of a protein in cells treated with doxycycline (+DOX) to the amount from untreated cells (−DOX), as determined by tandem mass tag mass spectrometry following streptavidin affinity purification. Proteins in green are previously described binding partners of Nesprin-1, whereas proteins in red are known centrosomal proteins investigated in this study. See also Figure S1 and Data S1. (E) Dual-color SD-*d*STORM image of Pericentrin (Pcnt, green) and Nesprin-1 (red, MANNES1E) [15, 18] at the nuclear surface of a differentiated C2C12 myoblast (left) and the same SD-*d*STORM image rendered to the resolution of a conventional wide-field microscope (right). Insets show higher magnifications of colocalization regions (arrows). The scale bar represents 1 μm. The scale bar of insets represents 100 nm. (F) Representative epi-fluorescence images of 48 hr differentiated C2C12 myotubes, transfected with the indicated siRNAs. Cells were stained for Pericentrin (Pcnt, red), nuclei (DAPI, blue), and myosin heavy chain (MHC, green) to identify myotubes. The scale bar represents 20 μm. See also Figures S2A–S2F. (G) Quantification of Pericentrin recruitment to the NE in myotube nuclei after treatment with the indicated siRNAs. Error bars ± SD; n represents total number of nuclei from at least three independent experiments. ^∗∗∗^p < 0.001; ^∗^p < 0.05; n.s., not statistically significant; t test. (H) Representative epi-fluorescence images of 72 hr differentiated primary myotubes from wild-type, *Sun1*^*−/−*^, *Sun2*^*−/−*^, or *Sun1*^*−/−*^;*Sun2*^*−/−*^ knockout mice, stained for Pericentrin (Pcnt, red), MHC (green), and nuclei (DAPI, blue). The scale bar represents 20 μm. (I) Quantification of Pericentrin recruitment to the NE as shown in (H). Error bars ± SD; n represents total number of nuclei from two independent experiments. ^∗∗^p < 0.01; n.s., not statistically significant, t test.

During skeletal muscle differentiation, Nesprin-1 expression switches from giant to smaller isoforms, particularly Nesprin-1α [17, 18]. Using C2C12 myoblasts that differentiate into myotubes upon serum withdrawal [19], we confirmed the upregulation of Nesprin-1α during myogenic differentiation (Figure 1A). To carry out the biotin identification (BioID) screen [13, 14], mycBirA^∗^ fused to Nesprin-1α (BioID-Nesprin-1α) was stably expressed in C2C12 cells and demonstrated to properly localize to the nuclear envelope (NE) upon doxycycline (DOX) induction (Figures 1B and 1C). BioID-Nesprin-1α expression was induced in the presence of biotin in proliferating C2C12 myoblasts and differentiated myotubes. Following affinity purification of biotinylated proteins on streptavidin-conjugated beads, we confirmed expression and biotinylation of BioID-Nesprin-1α and biotinylation of endogenous proteins (Figure S1A). Untreated cells (−DOX −biotin) and cells treated with only biotin (−DOX +biotin) were used as controls. Following tandem mass tag mass spectrometry and normalization to varying bait levels across samples, we identified 446 proteins preferentially associated with Nesprin-1α in myotubes compared to myoblasts, in at least two out of three experiments (Figure 1D; Data S1). These included known interactors, such as muscle A-kinase anchoring protein (mAkap; Akap6), kinesin light chains 1 and 2 (Klc1/2), and Kif5b [8, 20]. Nesprins, including Nesprin-1α/Nesprin-1, are anchored to the NE through interaction of its C-terminal KASH (Klarsicht/ANC-1/SYNE homology) domain with inner nuclear membrane SUN (Sad1/UNC-84) domain proteins, Sun1 and Sun2 [21]. We confirmed that Nesprin-1α/Nesprin-1 localization at the NE depends on Sun1/Sun2 (Figure S1C). In addition, we showed by small interfering RNA (siRNA)-mediated depletion that Klc1/2 localization at the NE depended on Nesprin-1 and both Sun1 and Sun2, as previously reported (Figures S1B–S1D) [8, 22].

Four centrosomal proteins (Akap450, Pcm1, Cep170, and Pericentrin) were preferentially enriched in myotube BioID-Nesprin-1α samples (Figure 1D; Data S1). Akap450, Pcm1, Pericentrin, Cdk5rap2, and γ-tubulin are centrosomal proteins reported to relocalize to the nucleus during skeletal muscle formation [1, 2, 3]. Concomitantly, microtubule (MT) nucleation activity is found at the NE, and the MT network itself is dramatically reorganized into dense bundles parallel to the long axis of differentiated myotubes [4, 23, 24]. Depletion of Nesprin-1 was previously reported to result in the loss of Pericentrin, Pcm1, and γ-tubulin from myotube nuclei by an unknown mechanism [5]. Our BioID data led us to hypothesize that the muscle-specific Nesprin-1α isoform [17] is the elusive molecular receptor for centrosomal proteins and for MT nucleation activity at the NE during skeletal muscle formation. Consistently, Nesprin-1α/Nesprin-1 and Pericentrin were found in close proximity at the NE of differentiated C2C12 myoblasts in spectral demixing direct stochastic optical reconstruction microscopy (SD-*d*STORM) [25, 26] with a lateral resolution of 20–35 nm (Figure 1E; SD-*d*STORM, insets). Note that the signals for Nesprin-1α/Nesprin-1 and Pericentrin overlap when the SD-*d*STORM data are rendered at the approximate resolution of a conventional wide-field microscope (Figure 1E; wide-field, insets).

### Nesprin-1α/Nesprin-1 Recruits Centrosomal Proteins to the NE Independently of Its Kinesin-1-Binding Domain

**Figure 2:**
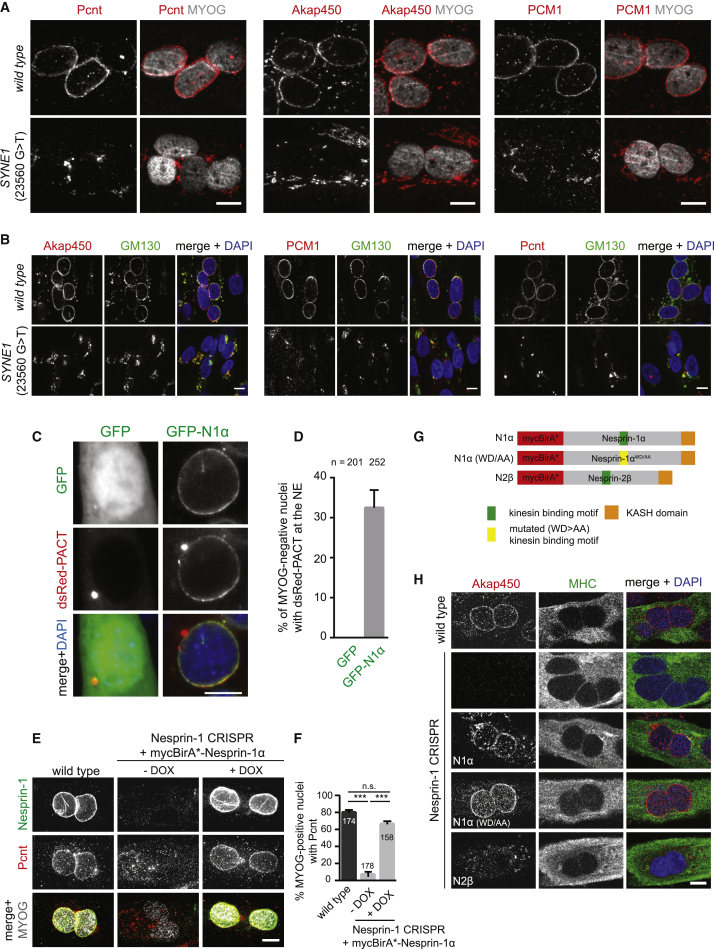
The Muscle-Specific Nesprin-1α Isoform Is Required for Recruiting Centrosomal Proteins to the Nucleus (A and B) Representative epi-fluorescence images of differentiated human immortalized myotubes from a healthy control (wild-type) or from a patient carrying a nonsense mutation within the *SYNE1* (23560 G>T) gene immunostained for Pericentrin (Pcnt, red), Akap450 (red), or PCM1 (red) and (A) Myogenin (MYOG, gray) as differentiation marker or (B) the *cis*-Golgi marker GM130 (green) and nuclei (DAPI, blue). The scale bar represents 10 μm. See also Figure S2G. (C) Representative epi-fluorescence images of C2C12 myoblasts transfected with dsRed-PACT and GFP or GFP-Nesprin-1α (GFP-N1α). Cells were stained for nuclei (DAPI, blue) and Myogenin (not shown). The scale bar represents 10 μm. (D) Quantification of dsRed-PACT recruitment to the NE in non-differentiated, Myogenin-negative C2C12 cells expressing GFP or GFP-Nesprin-1α. Error bars ± SD; n represents total number of nuclei from three independent experiments. (E) C2C12 wild-type or Nesprin-1 CRISPR mutant cells transduced with mycBirA^∗^-Nesprin-1α without and with 1 μg/mL doxycycline (−/+DOX) were differentiated for 48 hr, fixed, and stained for Nesprin-1 (green, clone 9F10), Pericentrin (Pcnt, red), and Myogenin (MYOG, gray). The scale bar represents 10 μm. See also Figures S2H–S2J. (F) Quantification of Pericentrin recruitment to the NE in Myogenin-(MYOG)-positive nuclei as described in (E). Error bars ± SEM; n represents total number of nuclei from three independent experiments. ^∗∗∗^p < 0.001; n.s., not statistically significant, Tukey’s multiple comparisons test following one-way ANOVA. (G) Schematic representation of the different myc-BirA^∗^-Nesprin constructs used for the experiments shown in (H). (H) C2C12 wild-type, untransduced Nesprin-1 CRISPR mutant cells or CRISPR mutant cells transduced with mycBirA^∗^-Nesprin-1α (N1α), mycBirA^∗^-Nesprin-1α with the LEWD motif mutated to LEAA (N1α [WD/AA]), or mycBirA^∗^-Nesprin-2β (N2β) were incubated with doxycycline and differentiated for 48 hr, fixed, and stained for myosin heavy chain (MHC, green), Akap450 (red), and nuclei (DAPI, blue). The scale bar represents 10 μm. See also Figure S2K.

We next depleted Nesprin-1α/Nesprin-1 in C2C12 myoblasts (Figure S2B) and analyzed Pericentrin localization in myotubes. As expected, Pericentrin was absent from the NE and dispersed within the cytoplasm in Nesprin-1-depleted myotubes (Figure 1F), with the loss of NE Pericentrin being similar to Pericentrin depletion (Figures 1G, S2A, and S2C). Protein levels of Pericentrin were unaffected by Nesprin-1 depletion during myogenic differentiation (Figure S2F). Pericentrin and Akap450 were also absent from the NE of a CRISPR/Cas9-edited clonal C2C12 cell line lacking NE-associated Nesprin-1 (Figures 2E, 2F, S2H, and S2I; −DOX samples). Curiously, the centriole protein Cep170 [27] that we also identified to be enriched in myotube BioID-Nesprin-1α samples localized to the NE even in the absence of Nesprin-1 (Figure S1E), suggesting that Nesprin-1-dependent and independent mechanisms might be involved in recruitment of centrosomal proteins to the NE.

Following co-depletion of Sun1 and Sun2, the percentage of nuclei showing Pericentrin at the NE was reduced to a similar extent as for Nesprin-1 or Pericentrin knockdown (Figures 1G, S2A, S2D, and S2E). Sun1 depletion alone somewhat reduced Pericentrin at the NE, whereas Sun2 depletion showed no effect (Figures 1G and S2A). Consistent with our RNAi data, *Sun1*^*−/−*^;*Sun2*^*−/−*^ mouse primary myoblasts differentiated to myotubes *in vitro* lacked Pericentrin at the NE; instead, Pericentrin was mislocalized to the cytoplasm (Figures 1H and 1I). This agrees with previous results demonstrating that only loss of both Sun1 and Sun2 affects Nesprin-1 nuclear localization in skeletal muscle [28]. However, *Sun1*^*−/−*^ myotubes appeared to have less Pericentrin at the NE than *Sun2*^*−/−*^ or wild-type myotubes, indicating that Sun1 might be the dominant SUN domain protein involved in Pericentrin NE recruitment during myogenic differentiation. Overall, we conclude that linker of nucleoskeleton and cytoskeleton (LINC) complexes comprising Nesprin-1α/Nesprin-1 and Sun1/2 are required for Pericentrin recruitment to the NE in myotubes.

Several NE proteins, including LINC complex components, are mutated in striated muscle diseases, like Emery-Dreifuss muscular dystrophy (EDMD) [29, 30, 31, 32, 33]. Recently, a homozygous nonsense mutation within the *SYNE1* gene (*2*3560 G>T) that encodes Nesprin-1 was identified in a congenital muscular dystrophy (CMD) patient [16], resulting in decreased mRNA expression of both Nesprin-1 giant and Nesprin-1α [15]. We asked whether this premature stop mutation within *SYNE1* affected NE localization of centrosomal proteins. First, we confirmed that Nesprin-1 isoforms were absent from the NE of immortalized myotubes from this CMD patient (*SYNE1* [23560 G>T]) but were present in a healthy control (Figure S2G). Next, we found that Pericentrin, Akap450, PCM1, and the *cis*-Golgi marker protein GM130 colocalized at the NE of healthy control myotubes (Figures 2A and 2B). In contrast, these centrosomal proteins were mislocalized to the cytoplasm in *SYNE1* (23560 G>T) patient myotubes, where they often concentrated at perinuclear regions together with mislocalized GM130-positive Golgi fragments. These results indicate that the aggregates of centrosomal proteins observed in Nesprin-1-depleted cells are retained at perinuclear Golgi complex fragments.

We next sought to ascertain whether the Nesprin-1α isoform was sufficient to recruit centrosomal proteins to the NE in non-differentiated cells via heterologous expression of Nesprin-1α and the Pericentrin-Akap450 centrosomal targeting (PACT) domain [34]. Non-differentiated C2C12 myoblasts were co-transfected with dsRed-PACT and GFP-Nesprin-1α or GFP. Only non-differentiated myoblasts (negative for Myogenin; not shown) were examined. Remarkably, dsRed-PACT was recruited to the NE in addition to the centrosome in about 30% of GFP-Nesprin-1α-expressing C2C12 cells but never found at the NE of GFP-expressing cells (Figures 2C and 2D). These data suggest that Nesprin-1α can already induce NE localization of centrosomal proteins in non-differentiated cells but that additional factors might be required to trigger the full recruitment process during myogenic differentiation. To determine whether Nesprin-1α is truly sufficient for NE localization of centrosomal proteins in differentiated myotubes, we transduced the C2C12 cell line lacking NE-associated Nesprin-1 with the same doxycycline-inducible mycBirA^∗^-Nesprin-1α construct used in the BioID experiments (Figure 1B) and examined Myogenin-positive cells for localization of Pericentrin and Akap450. Without doxycycline, Pericentrin and Akap450 were found within the cytoplasm in differentiated mycBirA^∗^-Nesprin-1α-transduced CRISPR cells (Figures 2E, 2F, S2H, and S2I). Induction of NE-localized mycBirA^∗^-Nesprin-1α by doxycycline led to recruitment of Pericentrin and Akap450 to the NE. Following washout of doxycycline, mycBirA^∗^-Nesprin-1α and Pericentrin were concomitantly lost from the NE after 5–7 days (Figure S2J). These data strongly suggest that Nesprin-1α is necessary and sufficient for NE localization of a subset of centrosomal proteins during skeletal muscle formation. Whether Nesprin-1α fulfils this function exclusively would require more comprehensive testing of the multitude of KASH-containing Nesprin isoforms [17, 35]. These results also demonstrate that mycBirA^∗^-Nesprin-1α used in the BioID experiments is biologically functional, thus strengthening our confidence in the physiological relevance of the BioID results.

Kinesin-1 is recruited to the NE via interaction with the LEWD kinesin-binding motif of Nesprin-1/Nesprin-2, and a WD/AA mutation in this domain disrupts the Nesprin-kinesin-1 interaction [8]. We set out to determine whether this domain was involved in the recruitment of Akap450 to the NE. We found that both wild-type and mutated Nesprin-1α (WD/AA) were competent to recruit Akap450 to the NE, when Nesprin-1 CRISPR myotubes were transduced with the respective doxycycline-inducible mycBirA^∗^-tagged Nesprin-1α or Nesprin-1α (WD/AA) constructs (Figures 2G and 2H). In contrast, the paralogous kinesin-binding Nesprin-2β, usually not expressed in C2C12 myotubes [17, 36], was not able to recruit Akap450 to the NE (Figure 2H). As expected, kinesin-1 NE localization was lost in Nesprin-1 CRISPR myotubes (Figure S2K). However, NE localization of kinesin-1 could be rescued upon expression of Nesprin-1α and Nesprin-2β, but not Nesprin-1α (WD/AA). Thus, Akap450 recruitment to the NE occurs independently of the kinesin-binding site of Nesprin-1α and is probably mediated by a region in Nesprin-1α that is absent in Nesprin-2β.

### Nesprin-1-Containing LINC Complex Recruits Akap450 to Nucleate MTs at the NE

**Figure 3:**
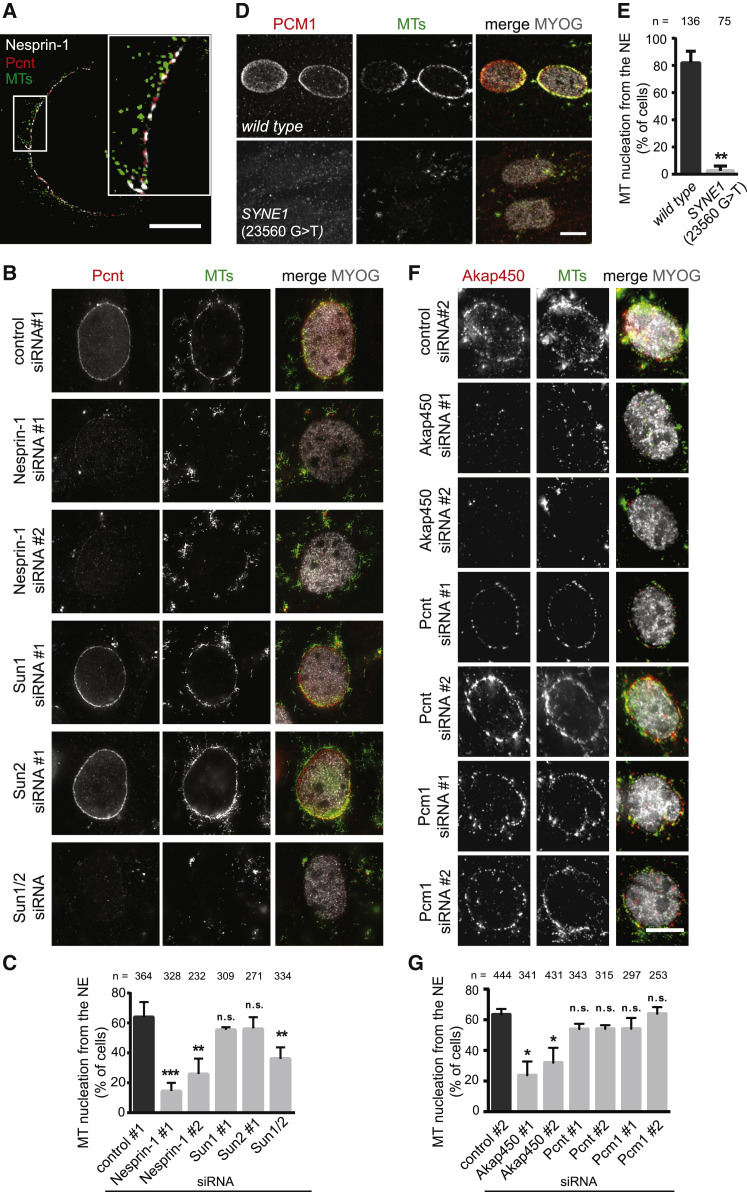
MT Nucleation from the NE Requires Nesprin-1, Sun1/2, and Akap450 (A) 3D-SIM fluorescent image of the nucleus of a differentiated C2C12 cell, stained for Nesprin-1 (white, MANNES1E), Pericentrin (Pcnt, red), and microtubules (MTs, green) after 5 min nocodazole washout to allow MT regrowth. The scale bar represents 5 μm. (B) 48 hr differentiated C2C12 cells, treated with the indicated siRNAs, were immunostained for Pericentrin (Pcnt, red), microtubules (MTs, green) and Myogenin (MYOG, gray) after nocodazole washout. The scale bar represents 10 μm. See also Figure S3A. (C) Quantification of the mean percentage (%) of Myogenin-positive cells with MT nucleation from the NE as described in (B). Error bars ± SD; n represents total number of nuclei from at least three independent experiments. ^∗∗∗^p < 0.001; ^∗∗^p < 0.01; n.s., not statistically significant, t test. (D) Differentiated human immortalized myotubes from a healthy control (wild-type) or from a patient carrying a nonsense mutation within the *SYNE1* gene (23560 G>T) were immunostained for PCM1 (red), microtubules (MTs, green) and Myogenin (MYOG, gray) after nocodazole washout. Images represent maximum projections of confocal z sections. The scale bar represents 10 μm. See also Figures S3B and S3C. (E) Quantification of the mean percentage (%) of Myogenin-positive cells with MT nucleation from the NE as described in (D). Error bars ± SD; n represents total number of nuclei from two independent experiments. ^∗∗^p < 0.01; t test. (F) 48 hr differentiated C2C12 cells, treated with the indicated siRNAs were immunostained for Akap450 (red), microtubules (MTs, green) and Myogenin (MYOG, gray) after nocodazole washout. The scale bar represents 10 μm. (G) Graph shows the mean percentage (%) of Myogenin-positive cells with MT nucleation from the NE as described in (F). Error bars ± SEM; n represents total number of nuclei from two independent experiments. ^∗^p < 0.05; n.s., not statistically significant, one-way ANOVA with Dunnett’s multiple comparisons test.

The recruitment of centrosomal proteins to the NE has been proposed to be responsible for MT nucleation from the nucleus in differentiated myoblasts and myotubes [4]. Our data suggest a requirement for LINC complexes in centrosomal protein recruitment to the NE. We thus interrogated which centrosomal components are involved in MT nucleation from the NE. We first used 3D structured illumination microscopy (3D SIM) in differentiated C2C12 myoblasts to show that Pericentrin is localized to the outer nuclear membrane (ONM) close to Nesprin-1α/Nesprin-1-containing foci from which MTs emanate (Figure 3A). To further explore these sites of MT nucleation, we used nocodazole to depolymerize MTs and then monitored MT regrowth in Myogenin-positive cells after nocodazole washout. Within five minutes, most of control siRNA-treated cells showed MT regrowth from the NE (Figures 3B and 3C). Additionally, we noticed MT regrowth from Pericentrin-positive seeds within the cytoplasm. In cells transfected with Nesprin-1 siRNA, we observed a strong reduction in MT regrowth from the NE, whereas MTs still regrew from Pericentrin-positive cytoplasmic seeds. In the absence of both Sun1/2, we also detected a reduction of MT regrowth from the NE, whereas depletion of Sun1 or Sun2 alone had little or no effect on regrowth. None of these siRNA-mediated depletions had an effect on the longitudinal MT array in untreated differentiated myoblasts when compared to control siRNA conditions (Figure S3A, untreated). Moreover, we confirmed that, in the presence of nocodazole, MTs were completely depolymerized and Pericentrin localization was unaffected after nocodazole treatment in all conditions (Figure S3A, nocodazole). Taken together, these results confirm that LINC complexes are required for MT nucleation from the NE, but not for the longitudinal alignment of MTs.

**Figure 4:**
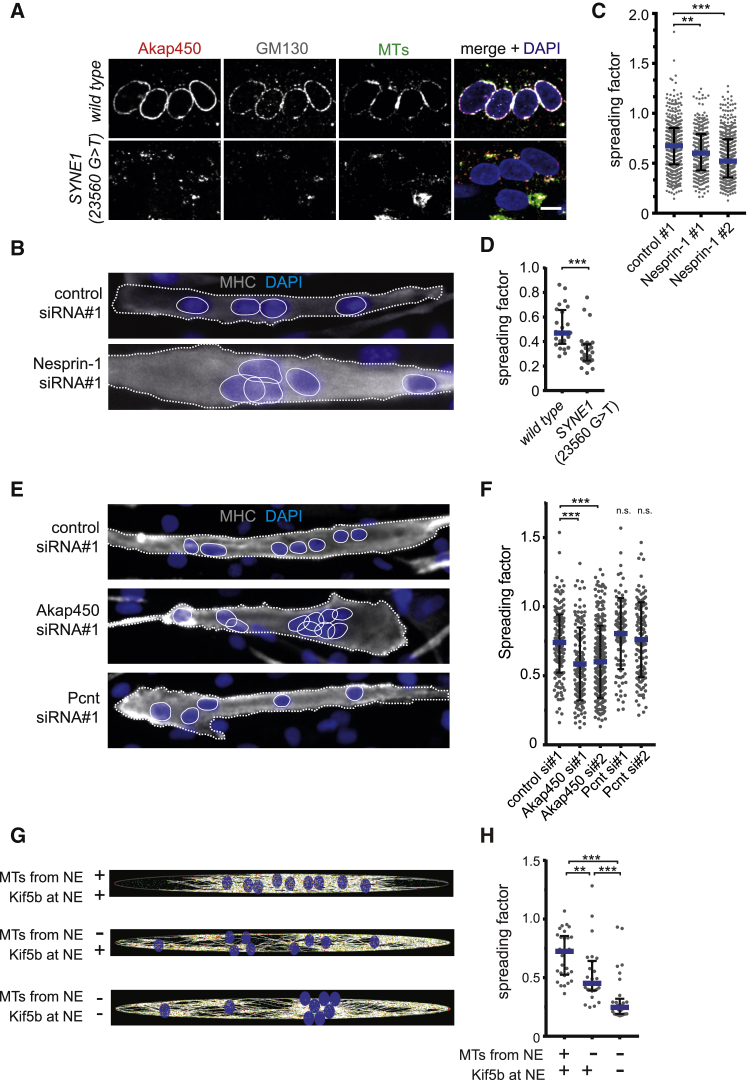
Microtubule Nucleation from the Nucleus Is Required for Proper Nuclear Positioning (A) Differentiated human immortalized myotubes from a healthy control (wild-type) or from a patient carrying a nonsense mutation within the *SYNE1* (23560 G>T) gene were immunostained with Akap450 (red), GM130 (gray), microtubules (MTs, green), and nuclei (DAPI, blue) following nocodazole washout. The scale bar represents 10 μm. See also Figure S4A. (B) C2C12 cells were transfected with the indicated siRNAs, differentiated for 48 hr, and stained for nuclei (DAPI, blue) and myosin heavy chain (MHC, white). Myotube outlines are marked by dashed lines, and nuclei are encircled. (C) Spreading factor analysis of nuclei in C2C12 myotubes as shown in (B) and for cells transfected with Nesprin-1 siRNA no. 2. Results are depicted as mean (blue line) with interquartile range (black bars) from three independent experiments. ^∗∗∗^p < 0.001; ^∗∗^p < 0.01; Mann-Whitney test. (D) Spreading factor analysis of nuclei in differentiated human immortalized myotubes from a healthy control (wild-type) or *SYNE1* (23560 G>T) patient cells. Results are depicted as mean (blue line) with interquartile range (black bars) from two independent experiments. ^∗∗∗^p < 0.001; Mann-Whitney test. See also Movie S1. (E) C2C12 cells were transfected with the indicated siRNAs, differentiated for 48 hr, and stained for nuclei (DAPI, blue) and myosin heavy chain (MHC, white). Myotube outlines are marked by dashed lines, and nuclei are encircled. (F) Spreading factor analysis of nuclei in C2C12 myotubes shown in (E) and for myotubes transfected with Akap450 siRNA no. 2 and Pcnt (Pericentrin) siRNA no. 2. Results are depicted as mean (blue line) with interquartile range (black bars) from four independent experiments. ^∗∗∗^p < 0.001; n.s., not statistically significant, Mann-Whitney test. (G) Snapshots of myotubes simulated with Cytosim. Nuclei are blue, and MTs in white. Three conditions are shown from top to bottom, with (+) and without (−) MTs nucleated from the NE and ±Kif5b anchored at the NE, as indicated. ^∗∗∗^p < 0.001; Mann-Whitney test. See also Movie S2 and Figures S4B–S4D. (H) Spreading factor analysis of nuclei in simulated myotubes as shown in (G). Results are depicted as mean (blue line) with interquartile range (black bars). ^∗∗∗^p < 0.001; ^∗∗^p < 0.01; Mann-Whitney test. See also Figure S4E.

To corroborate these findings, we examined MT nucleation from the NE using the same MT regrowth assay in *SYNE1* (23560 G>T) patient cells. Wild-type myotubes predominantly nucleated MTs from the NE but also from minor PCM1- or Akap450-positive seeds in the cytoplasm (Figures 3D, 3E, and 4A). In contrast, in almost all *SYNE1* (23560 G>T) patient myotubes, all MT nucleation involved PCM1- or Akap450-positive seeds within the cytoplasm with little or no MT regrowth occurring from the NE. Nevertheless, myotubes from *SYNE1* (23560 G>T) patient cells continued to maintain longitudinal MT arrays, comparable to those observed in wild-type myotubes (Figure S3B, untreated). Additionally, treatment of wild-type or *SYNE1* (23560 G>T) cells with nocodazole completely depolymerized MTs with little effect on PCM1 distribution, be it NE-associated or cytoplasmic (Figure S3B, nocodazole). *SYNE1* (23560 G>T) patient myotubes only displayed MT nucleation defects but no obvious disorganization of the actin cytoskeleton, consistent with Nesprin-1α lacking the N-terminal actin-binding domain of Nesprin-1 giant (Figure S3C). Taken together, these results imply that Nesprin-1 occupies a central position in MT nucleation from the NE, most likely via the recruitment of key centrosomal components.

The identity of this key MT-nucleating component at the NE is currently unknown. Our BioID experiments identified two centrosomal proteins, Pericentrin and Akap450, which are known to be involved in MT nucleation at the centrosome through the recruitment of γ-tubulin ring complexes (γ-TuRCs) [37, 38]. We also identified Pcm1, which is thought to indirectly impact on MT organization through the recruitment of other proteins to the centrosome [39]. To determine whether any of these proteins is required for MT nucleation from the NE, MT regrowth experiments were carried out following depletion of Pericentrin, Akap450, or Pcm1 using two different siRNAs targeting each of the mRNAs. In cells treated with Pericentrin or Pcm1 siRNA, MTs regrew from nuclei to a similar extent as observed for control siRNA-treated cells (60% of Myogenin-positive cells). In contrast, in cells treated with Akap450 siRNA, MTs regrew from less than 30% of Myogenin-positive nuclei (Figures 3F and 3G). These data suggest that Akap450, but not Pericentrin, is the dominant receptor for MT-nucleating γ-TuRC complexes at the NE. Consistently, in myofibers isolated from mouse skeletal muscle, Akap450 remained exclusively at the NE, whereas Pericentrin was also found at MT-nucleating Golgi elements [40].

To elucidate the role of centrosomal proteins on the recruitment of other centrosomal proteins, we depleted Akap450, Pericentrin, Pcm1, and Cdk5rap2 (another pericentriolar material protein) [41] using specific siRNAs and examined their NE localization (Figure S4A). We found that depletion of Pericentrin, Pcm1, and Cdk5rap2 did not affect NE localization of Akap450. Pcm1 also localized to the NE independently of the other centrosomal proteins studied here. In contrast, Pericentrin NE localization was partially reduced by Pcm1 depletion as previously shown [5, 39]. Consistent with previous findings at the centrosome, Cdk5rap2 localization depended on Pericentrin [42, 43, 44]. These results further support the unique role of Akap450 on MT nucleation from the NE and suggest that the recruitment of a subset of centrosomal proteins requires interdependent interactions.

### MT Nucleation from the Nucleus Is Necessary for Proper Nuclear Positioning

Correct nuclear positioning is important for muscle function, and mislocalized nuclei are often associated with muscular diseases [45, 46, 47]. The MT network, MT motor proteins, and MT-associated proteins (MAPs) have been implicated in nuclear positioning during skeletal muscle formation [8, 22, 46, 47, 48]. Furthermore, Nesprin-1 was reported to be involved in the distribution of skeletal muscle nuclei through the recruitment of kinesin-1 motor proteins to the NE *in vivo* and *in vitro* [8, 9, 10, 11, 12, 49]. However, it is unknown whether MT nucleation from the NE is also important for nuclear positioning. To confirm the role of Nesprin-1 in nuclear positioning [8], we differentiated C2C12 cells treated with control or Nesprin-1*-*targeting siRNAs and determined the nuclear distribution along myotubes by calculating the nuclear spreading factor (SF), defined as the ratio between the observed and the maximal theoretical average distance between nuclei according to the myotube length. Nuclei in Nesprin-1-depleted myotubes were less spread throughout the myotube when compared to control myotubes (Figures 4B and 4C). Similarly, we observed a reduction of nuclear spreading in *SYNE1* (23560 G>T) patient myotubes when compared to wild-type myotubes (Figure 4D; Movie S1). If MT nucleation from the NE is required for nuclear positioning, then removing Akap450 should also induce nuclear clustering in myotubes. Consistently, depletion of Akap450, but not Pericentrin significantly reduced the SF (Figures 4E and 4F). These results strongly support the hypothesis that Nesprin-1-mediated MT nucleation from the NE via Akap450 is required for nuclear positioning in myotubes.

To determine the role of MT nucleation from the NE on nuclear positioning, we developed a computational model of nuclear movement in myotubes, using the cytoskeleton simulation engine Cytosim [50]. All simulation parameters were set using measured values when possible or varied within reasonable ranges (Table S1; Figures S4B–S4D). Briefly (see STAR Methods for details), in control situations, nuclei featured MT-nucleating complexes at the NE and MT plus-end (Kif5b) and MT minus-end (dynein) directed motor proteins. Moreover, we incorporated MAP4 and the Kif5b-MAP7 complex within the myotube cytoplasm to accomplish anti-parallel MT organization and sliding [47, 51]. Using these minimal settings, we simulated nuclear movements within *in silico* myotubes and observed that nuclei moved dynamically and became evenly dispersed along their long axes. These movements *in silico* recapitulated those observed in cultured myotubes (Figures 4G, 4H, and S4E; Movie S2). Next, we removed MT nucleation activity from the NE and added it to centrosome-like structures randomly positioned in the cytoplasm, as observed in Nesprin-1-depleted myotubes in vitro. No other features were changed, including the NE localization of the plus-end motors (Kif5b). In this situation, nuclei clustered in the middle of the myotube or in small groups throughout the myotube, leading to a significantly decreased SF (Figures 4G, 4H, and S4E; Movie S2). Finally, we simulated nuclear distribution in myotubes where we removed both MT nucleation activity and plus-end motors (Kif5b) from the NE. We found that nuclei further clustered together and the SF was decreased even more, when compared to removal of MT nucleation alone (Figures 4G, 4H, and S4E; Movie S2). Thus, Nesprin-1-mediated MT nucleation from the NE via Akap450 has a role in nuclear positioning in myotubes independent of the recruitment of plus-end motor proteins to the NE. However, only the combined action of Nesprin-1-mediated MT nucleation and Kif5b/kinesin-1 recruitment to the nucleus seems to result in proper nuclear positioning in myotubes.

In this work, we identified the muscle-specific Nesprin-1α isoform to be required for the recruitment of several centrosomal proteins to the NE during skeletal muscle formation. Among the recruited centrosomal proteins, only Akap450 was involved in regulating MT nucleation from the NE. Our modeling and Akap450 depletion experiments indicated that MT nucleation is required for proper nuclear positioning in addition to NE-localized kinesin-1 motor protein activity. The finding that Akap450 recruitment is independent of kinesin-1 NE localization raises the intriguing possibility of synergistic coordination of MT nucleation with MT motor activity on individual Nesprin-1α/Nesprin-1 molecules to facilitate efficient nuclear positioning. Future work will address whether these two different Nesprin-1 activities work within the same LINC complex containing Nesprin-1α and Sun1/2 or whether this activity is segregated.

## STAR★Methods

### Key Resources Table

**Table:** 

REAGENT or RESOURCE	SOURCE	IDENTIFIER
**Antibodies**		

Rabbit polyclonal anti-Pericentrin	Covance	Cat# PRB-432C; RRID: AB_2313709
Rabbit polyclonal anti-Pericentrin	Abcam	Cat#ab4448; RRID: AB_304461
Mouse monoclonal anti-Pericentrin	BD Biosciences	Cat#611814; RRID: AB_ 399294
Rabbit anti-Pericentrin	Kunsoo Rhee [52]	N/A
Mouse monoclonal anti-myosin, sarcomere (MHC)	Developmental Studies Hybridoma Bank (DSHB)	Cat#MF20; RRID: AB_ 2147781
Mouse monoclonal anti-GAPDH [GT239]	GeneTex	Cat#GTX627408; RRID: AB_11174761
Mouse monoclonal anti-Nesprin-1, clone 9F10	This paper	N/A
Mouse monoclonal anti-Nesprin-1, clone MANNES1A	Glenn E. Morris [18]	N/A
Mouse monoclonal anti-Nesprin-1, clone MANNES1E	Glenn E. Morris [18]	N/A
Mouse monoclonal anti-Myc, 9E10	Developmental Studies Hybridoma Bank (DSHB)	Cat#9E10; RRID: AB_ 2266850
Rabbit polyclonal anti-Akap9	Sigma-Aldrich	Cat#HPA026109; RRID: AB_844688
Rabbit polyclonal anti-PCM1	Bethyl Laboratories	Cat#A301-149A; RRID: AB_ 2160197
Mouse monoclonal anti-Myogenin, F5D	Developmental Studies Hybridoma Bank (DSHB)	Cat#F5D; RRID: AB_2146602
Rabbit polyclonal anti-Sun1 (UNC84A)	Sigma-Aldrich	Cat#AV49929; RRID: AB_1858623
Rabbit anti Sun1	[53]	N/A
Rabbit anti-Sun2	ImmuQuest	Cat# IQ444; RRID: AB_10659143
Rabbit monoclonal anti-Kif5b [EPR10276(B)]	Abcam	Cat#ab167429
anti-Klc1/2	Scott T. Brady	N/A
Mouse monoclonal anti-Cep170	Thermo Fisher Scientific	Cat#41-3200; RRID: AB_ 2533502
Mouse monoclonal anti-GM130	BD Biosciences	Cat##610823; RRID: AB_398142
Rabbit polyclonal anti-CDK5RAP2	Bethyl Laboratories	Cat#IHC-00063; RRID: AB_2076863
Donkey anti-Rabbit IgG (H+L) Highly Cross-Adsorbed Secondary Antibody, Alexa Fluor 488	Thermo Fisher Scientific	Cat#A21206
Donkey anti-Rabbit IgG (H+L) Highly Cross-Adsorbed Secondary Antibody, Alexa Fluor 568	Thermo Fisher Scientific	Cat#A10042
Goat anti-Mouse IgG1 Cross-Adsorbed Secondary Antibody, Alexa Fluor 488	Thermo Fisher Scientific	Cat#A21121
Goat anti-Mouse IgG (H+L) Cross-Adsorbed Secondary Antibody, Alexa Fluor 555	Thermo Fisher Scientific	Cat#21424
Goat anti-Mouse IgG1 Cross-Adsorbed Secondary Antibody, Alexa Fluor 568	Thermo Fisher Scientific	Cat#A21124
Goat anti-Mouse IgG1 Cross-Adsorbed Secondary Antibody, Alexa Fluor 647	Thermo Fisher Scientific	Cat#A21240
Goat anti-Mouse IgG2b Cross-Adsorbed Secondary Antibody, Alexa Fluor 568	Thermo Fisher Scientific	Cat#A21144
Goat anti-Rabbit IgG (H+L) Highly Cross-Adsorbed Secondary Antibody, Alexa Fluor 647	Thermo Fisher Scientific	Cat#21245
Goat anti-Mouse IgG2b Cross-Adsorbed Secondary Antibody, Alexa Fluor 647	Thermo Fisher Scientific	Cat#A21242
Donkey anti-Rat IgG (H+L) Highly Cross-Adsorbed Secondary Antibody, Alexa Fluor 488	Thermo Fisher Scientific	Cat#A21208
Goat anti-Mouse IgG (H+L) Highly Cross-Adsorbed Secondary Antibody, Alexa Fluor Plus 647 (for SD-*d*STORM)	Thermo Fisher Scientific	Cat#A32728
Goat anti-Rabbit IgG (H+L) Highly Cross-Adsorbed Secondary Antibody, Alexa Fluor Plus 647 (for SD-*d*STORM)	Thermo Fisher Scientific	Cat#A32733
Donkey anti-Mouse IgG (H+L) Highly Cross-Adsorbed Secondary Antibody, CF680 (for SD-*d*STORM)	Biotium	Cat#20483
Donkey anti-Rabbit IgG (H+L) Highly Cross-Adsorbed Secondary Antibody, CF680 (for SD-*d*STORM)	Biotium	Cat#20344

**Bacterial and Virus Strains**		

*Escherichia coli*: BL21(DE3) strain	Stratagene / Agilent	Cat#200131

**Chemicals, Peptides, and Recombinant Proteins**		

Nocodazole	Sigma-Aldrich	Cat#M1404
Lipofectamine 2000 transfection reagent	Thermo Fisher Scientific	Cat#11668019
Lipofectamine 3000 transfection reagent	Thermo Fisher Scientific	Cat#L3000008
jetPRIME	Polyplus-transfection	Cat#114-07
Biotin	Sigma-Aldrich	Cat#B4639
Doxycycline	Fisher Bioreagents	Cat#BP2653-1
Puromycin	Sigma-Aldrich	Cat#P8833
Lys-C protease	Wako	Cat# 129-02541
Trypsin	Promega	Cat#V5111
Dynabeads MyOne Streptavidin C1	Thermo Fisher Scientific	Cat#65001
Collagenase type II	GIBCO	Cat#17101-015
Dispase	GIBCO	Cat#17105-041
gelatin	Sigma-Aldrich	Cat#G1890
Matrigel	Corning Life Sciences	Cat#354230
Dulbecco’s modified Eagle’s medium (DMEM)	GIBCO	Cat#41966
DMEM with GlutaMAX	GIBCO	Cat#61965-026
DMEM 199 medium	GIBCO	Cat#41150
Iscove’s Modified Dulbecco’s Medium (IMDM) with GlutaMAX	GIBCO	Cat#31980
Hanks’ Balanced Salt Solution	GIBCO	Cat#14170-112
Ham’s F10	GIBCO	Cat#11550043
Advanced RPMI 1640	GIBCO	Cat#12633020
Opti-MEM I Reduced Serum Medium	GIBCO	Cat#31985070
Fetal calf serum (FCS)	Eurobio	Cat#CVFSVF0001
Fetal bovine serum (FBS)	GIBCO	Cat#10270
Penicillin/streptomycin	GIBCO	Cat#15140-122
Horse serum	GIBCO	Cat#26050088
HAT supplement	GIBCO	Cat#21060017
Goat serum	Sigma-Aldrich	Cat#G9023
Bovine fetuin	Life Technologies	Cat#10344026
recombinant human EGF	Life Technologies	Cat#PHG0311
recombinant human FGF-basic	Life Technologies	Cat#PHG0026
bFGF	Invitrogen	Cat#13256029
recombinant human insulin	Sigma-Aldrich	Cat#91077C
dexamethasone	Sigma-Aldrich	Cat#D4902
gentamicin	GIBCO	Cat#15750
Complete protease inhibitor	Roche	Cat#11697498001
β-mercaptoethylamine (MEA)	Sigma-Aldrich	Cat#30070
glucose oxidase	Sigma-Aldrich	Cat#G2133
catalase	Sigma-Aldrich	Cat#C100
Lysozyme	Sigma-Aldrich	Cat#L7651
Glutathione Sepharose 4B	GE Healthcare	Cat#17075601
GST-Nesprin-1α-326-634	This paper	N/A
Polyethylene glycol solution, 50% w/v, Mw ∼1500	Sigma-Aldrich	Cat#P7181
Freund’s Complete Adjuvant	Thermo Fisher Scientific	Cat#77140
Freund’s Incomplete Adjuvant	Thermo Fisher Scientific	Cat#77145
Fluoromount-G	Southern Biotech	Cat#0100-01
Prolong Diamond	Invitrogen	Cat#P36961
1,4-Diazabicyclo[2.2.2]octane (DABCO)	Sigma-Aldrich	Cat#D27802
Vectashield	Vector Laboratories	Cat#H-1000
4’,6-diamidino-2-phenylindole dihydrochloride (DAPI)	Molecular Probes	Cat#D1306
4x Laemmli sample buffer	Bio-Rad	Cat#1610747
1x Tris/Glycine buffer	Bio-Rad	Cat#1610771
IRDye 800CW Streptavidin	Li-Cor	Cat#925-32230
IRDye 680RD Goat anti-Mouse IgG (H + L)	Li-Cor	Cat#926-68070

**Critical Commercial Assays**		

ThermoScriptTM RT-PCR System for First-Strand cDNA Synthesis	Thermo Fisher Scientific	Cat#11146025
Pierce BCA Protein Assay Kit	Thermo Fisher Scientific	Cat#23225
TMT10plex Isobaric Label Reagent Set	Thermo Fisher Scientific	Cat# 90406
SuperSignal West Pico Chemiluminescence kit	Thermo Fisher Scientific	Cat#34080
Luminata Forte Western HRP Substrate	Millipore	Cat#WBLUF0100

**Experimental Models: Cell Lines**		

Mouse: C2C12 cell line	American Type Culture Collection (ATCC)	Cat# CRL-1772; RRID: CVCL_0188
Human: immortalized healthy control myoblasts	[15, 16]	N/A
Human: immortalized myoblasts from a congenital muscular dystrophy patient carrying a homozygous nonsense mutation within the *SYNE1 (23560 G>T)* gene	[15, 16]	N/A
Rat hybridoma cell line YL1/2, anti-tubulin reactivity	European Collection of Authenticated Cell Cultures (ECACC)	Cat#92092402; RRID: CVCL_J781
Human: HeLa cell line	American Type Culture Collection (ATCC)	Cat#: CCL-2 RRID: CVCL_0030
Rat: NRK cell line	American Type Culture Collection (ATCC)	Cat#: CRL-6509 RRID: CVCL_3758
Mouse: SP2/0-Ag14 myeloma cell line	Gift of Karl Riabowol [52]	RRID: CVCL_2199

**Experimental Models: Organisms/Strains**		

Mouse: Sun1^−/−^: *B6;129-Sun1*^*tm1.1Ktj*^*/N*	[54]	RRID: MGI:3838371
Mouse: Sun2^−/−^: B6;129S6-*Sun2*^*tm1Mhan*^/J	[28]	JAX: 012716 RRID: MGI:3850091

**Oligonucleotides**		

siRNA targeting sequence: mouse Nesprin-1 #1 CCAUCGAGUCUCACAUCAAtt	GeneCust	N/A
siRNA targeting sequence: mouse Nesprin-1 #2 AGUAAGAGGAGAAGGAAUAtt	GeneCust [8]	N/A
siRNA targeting sequence: mouse Sun1 #1 GGCUAUUGAUUCGCACAUUtt	Ambion	Cat#4390771 s94911
siRNA targeting sequence: mouse Sun2 #1 CUCUCAGGAUGAUAACGAUtt	Ambion	Cat#4390771 s104591
siRNA targeting sequence: mouse Pericentrin #1 GCCGAUCAACAAUUGCUAAtt	Ambion	Cat#4390771 s71317
siRNA targeting sequence: mouse Pericentrin #2 GGGUUUAAUGAAUUGGUCAtt	Ambion	Cat#4390771 s71316
siRNA targeting sequence: mouse Akap450 #1 AUCACUGUGCAACUUGAAUAAAGAA	Integrated DNA Technologies	Cat# mm.Ri.Akap9.13.1
siRNA targeting sequence: mouse Akap450 #2 UACCUUUCAUUGGACAGGUUUCUAUCG	Integrated DNA Technologies	Cat# mm.Ri.Akap9.13.2
siRNA targeting sequence: mouse Pcm1 #1 AGUCAGAUUCUGCAACAUGAUCUTG	Integrated DNA Technologies	Cat# mm.Ri.Pcm1.13.1
siRNA targeting sequence: mouse Pcm1 #2 AAUAGUAUCCCGUAAAGCUUCAAACAU	Integrated DNA Technologies	Cat# mm.Ri.Pcm1.13.2
See also Table S2 for other siRNAs and primers	N/A	N/A

**Recombinant DNA**		

pcDNA3.1 EGFP-Nesprin-1α	This paper	N/A
pTripZ-mycBirA^∗^-Nesprin-1α	This paper	N/A
pGEX-4T-1 GST-Nesprin-1α-326-634	This paper	N/A
pTripZ-mycBirA^∗^-Nesprin-2β	This paper	N/A
pTripZ-mycBirA^∗^-Nesprin-1α (WD/AA)	This paper	N/A
pX330-U6-Chimeric BB-CBh-hSpCas9	[55]	Addgene plasmid Cat#42230
pX330-Nesprin-1-N-ter-CRISPR	This paper	N/A
pX330-Nesprin-1-C-ter-CRISPR	This paper	N/A
dsRed-PACT	Sean Munro [34]	N/A

**Software and Algorithms**		

Cytosim	Francois Nedelec [50]	N/A
GraphPad Prism	GrahPad Software	Version 6; RRID: SCR_002798
Fiji	https://fiji.sc	RRID: SCR_002285
SoftWorX program	Applied Precision	N/A
Metamorph Software	Molecular Devices	RRID: SCR_002368
Proteome Discoverer 2.1	Thermo Fisher Scientific	RRID: SCR_014477
Mascot 2.5.1	Matrix Science	RRID: SCR_014322
Spreading factor algorithm	This paper, VBA Excel	N/A

**Other**		

Inverted Nikon Ti microscope	Nikon	N/A
DeltaVision CORE	GE / Applied Precision	N/A
Plan Apochromat 40 × /1.35 NA oil-immersion objective lens	Olympus	N/A
CCD camera CoolSNAP HQ	Photometrics	N/A
Leica SPE confocal microscope	Leica	N/A
Nikon Spinning disk confocal microscope	Nikon	N/A
OMX V4 Blaze	GE Healthcare	Cat#29065721
CoolSNAP HQ2 camera	Roper Scientific	N/A
Zeiss LSM510 confocal microscope	Carl Zeiss AG	N/A
Environmental chamber	Okolab	Stage Top Incubator, H301
Odyssey Imaging System	Li-Cor	No longer available
Orbitrap Fusion mass spectrometer coupled to nanoUPLC Easy LC 1000 system	Thermo Fisher Scientific	N/A
Sep-Pak C-18 cartridges	Waters	Cat# WAT051910
C18 Basic Resins 10 μm	Dr. Maisch	Cat# r10.b9.0025
Easy spray column 50 cm x 75 μm (C18, 1.8 μm)	Thermo Fisher Scientific	Cat#ES-803
8-well μ-slides	ibidi	Cat#80826
96-well μ-plates	ibidi	Cat#89626
Slides with molds providing a 100 μl spherical void	Carl Roth	Cat#H884.1
TetraSpeck multicolor beads	Invitrogen	Cat#T7279
QIAshredder	Quiagen	Cat#79656
4-15% Mini-PROTEAN TGX protein gels	Bio-Rad	Cat#4561084
Trans-Blot Turbo Transfer system	Bio-Rad	Cat# 1704150
4-12% NuPAGE NovexBis-Tris gels	Invitrogen	Cat#NP0335
Amersham Hyperfilm	GE Healthcare	Cat#10094984

### Contact for Reagent and Resource Sharing

Further information and requests for resources and reagents should be directed to and will be fulfilled by the Lead Contact, Edgar R. Gomes (edgargomes@medicina.ulisboa.pt).

### Experimental Model and Subject Details

#### Cell lines and culture

Mouse C2C12 myogenic precursor cells were cultivated at 37°C/5% CO_2_ in Dulbecco’s modified Eagle’s medium (DMEM) containing 4.5 g/L D-glucose, 4 mM L-glutamine, 1 mM sodium pyruvate,10% fetal calf serum, and 100 U/ml penicillin; 100 μg/ml streptomycin. C2C12 cells are female. Cells were not authenticated. For immunofluorescence, C2C12 myoblasts were seeded on coverslips, 8-well μ-slides or 96-well μ-plates coated with 0.1% gelatin or Matrigel diluted 1:100 in DMEM. When C2C12 cells reached ∼90% confluence, they were switched to DMEM supplemented with 2% horse serum and 100 U/ml penicillin; 100 μg/ml streptomycin to induce myogenic differentiation for the indicated time points.

For generating Nesprin-1 CRISPR cell lines, wild-type C2C12 were transfected overnight using Lipofectamine 3000 according to manufacturer’s instructions with pX330 encoding Nesprin-1 N- and C-termini-directed sgRNAs and pEGFP-N1 in a 9:9:2 ratio. Cells with high levels of EGFP expression, representing high levels of pX330 transfection, were sorted as single cells into 96-well plates at the Singapore Immunology Network FACS facility. Loss of Nesprin-1 NE localization in clonal lines was assessed by immunofluorescence microscopy. Nesprin-1 CRISPR cell lines were cultivated and maintained as wild-type C2C12 cells as described above.

Human myoblasts from a healthy control or from a congenital muscular dystrophy patient carrying a homozygous nonsense mutation within the *SYNE1 gene* (nucleotide 23560 G>T) were immortalized by Kamel Mamchaoui and Vincent Mouly (Center for Research in Myology, Paris, France) via transduction with retrovirus vectors expressing *hTERT* and *Cdk4* as described previously [15, 16]. Human myoblasts were cultivated in growth medium containing DMEM with GlutaMAX and DMEM 199 medium (4:1 ratio), supplemented with 20% FBS, 25 μg/ml bovine fetuin, 5 ng/ml recombinant human EGF, 0.5 ng/ml recombinant human FGF-basic, 5 μg/ml recombinant human insulin, 0.2 μg/ml dexamethasone, and 0.1% gentamicin. For immunofluorescence, human myoblasts were seeded on coverslips coated with Matrigel diluted 1:100 in DMEM, grown to ∼90% confluence and induced for differentiation by changing to Iscove’s Modified Dulbecco’s Medium (IMDM) with GlutaMAX, 2% horse serum, and 0.1% gentamicin.

#### Mouse strains and primary myoblasts

The Sun1^−/−^ (*B6;129-Sun1*^*tm1.1Ktj*^*/N* RRID: MGI:3838371) and Sun2^−/−^ (B6;129S6-*Sun2*^*tm1Mhan*^/J, JAX: 012716, RRID: MGI:3850091) mice were previously described [28, 54] and were maintained at the A^∗^STAR Biological Resource Centre facility in accordance with the guidelines of the Institutional Animal Care and Use Committee. Primary myoblasts were obtained from E18.5 mouse embryos as *Sun1*^*−/−*^ *Sun2*^*−/−*^ double mutant mice die shortly after birth. Limb muscles were dissected away from skin and bones. Muscle tissue was digested in enzyme solution (0.5% w/v collagenase type II, 1.2 U/ml dispase, 2.5 mM CaCl_2_, 25 mM HEPES in Hanks’ Balanced Salt Solution) for 30 min at 37°C [56] with gentle trituration using a plastic pipette tip every 10-15 min. Following addition of DMEM containing 10% FBS, resultant tissue slurry was filtered successively through 70 μm and 40 μm cell strainers. Cells were plated onto tissue culture plates for 1-2 hr in 37°C/5% CO_2_ to allow contaminating fibroblasts to adhere. The myoblast enriched fraction was then plated onto 0.1% gelatin-coated tissue culture plates and maintained in Ham’s F10 containing 20% fetal bovine serum and 10 μg/ml bFGF in 37°C/5%CO_2_. Myoblast enrichment by preplating of fibroblasts was repeated every 2-3 days until most fibroblasts were removed from the myoblast culture. Primary myoblasts were differentiated into myotubes by changing to DMEM supplemented with 2% horse serum and 100 U/ml penicillin; 100 μg/ml streptomycin. Due to the difficulty of sexing mouse embryos visually, sex of primary myoblasts derived from E18.5 embryos was not determined.

### Method Details

#### Antibodies

The following primary antibodies were used for immunofluorescence and Western Blot analysis: rabbit anti-Pericentrin, mouse anti-Pericentrin, mouse anti-myosin, sarcomere MHC, mouse anti-GAPDH, mouse anti-Nesprin-1 (clone 9F10), mouse anti-Myc (clone 9E10), rabbit anti-Akap9, rabbit anti-PCM1, mouse anti-Myogenin (clone F5D), rat anti-tyrosinated-α-tubulin (clone YL1/2), rabbit anti-Sun1, rabbit anti-Sun2, rabbit anti-Kif5b, mouse anti-Cep170, mouse anti-GM130, and rabbit anti-CDK5RAP2. The mouse anti-Nesprin-1 (MANNES1E; MANNES1A), anti-Klc1/2, and anti-Pericentrin antibodies were kind gifts from Glenn E. Morris, Scott T. Brady, and Kunsoo Rhee, respectively. Details of secondary antibodies are in the Key Resources Table.

#### siRNA/DNA transfection

C2C12 cells were transfected at approximately 30% confluency with siRNA (20 or 50 nM final concentration) by addition of transfection complexes pre-formed for 20 min, containing 0.3 μL Lipofectamine RNAiMAX per pmol of siRNA in Opti-MEM medium. The following siRNAs were used: non-targeting control (NC) siRNA#1, NC siRNA#2, NC siRNA#3, Nesprin-1 siRNA#1, Nesprin-1 siRNA#2, Nesprin-1 siRNA#3, Sun1 siRNA#1, Sun1 siRNA#2, Sun2 siRNA#1, Sun2 siRNA#2, Pericentrin (Pcnt) siRNA#1, Pcnt siRNA#2 or 1:1 mix of Pcnt siRNA#1 and #2, Akap450 siRNA#1, Akap450 siRNA#2, Pcm1 siRNA#1 and Pcm1 siRNA#2, Cdk5rap2 siRNA#1, and Cdk5rap2 siRNA#2, and their sequences are provided in the Key Resources Table and Table S2.

#### Plasmids

Mouse cDNA was obtained from mouse hindlimb skeletal muscle RNA using ThermoScript RT-PCR System for First-Strand cDNA Synthesis with random hexamers. Human cDNA was obtained from HeLa cell RNA using the same kit. Nesprin-1α cDNA was obtained by PCR from mouse skeletal muscle cDNA using the following primers 5′-GCGCCTCGAGATGGTGGTGGCAGAGGACTTGC-3′ and 5′-GCGCCTTAAGTCAGAGTGGAGGAGGACCGTT-3′ and ligated into pcDNA3.1 with an EGFP tag following restriction digest with XhoI and AflII. pTripZ-mycBirA^∗^-Nesprin-1α was obtained by PCR amplification of Nesprin-1α from pcDNA3.1 containing Nesprin-1α using 5′-AAGCTCGAGATGGTGGTGGCAGAGGACTTGC-3′ and 5′- AGGCCACGCGTCCTAGGTCAGAGTGGAGGAGG-3′ followed by restriction digestion with XhoI and MluI and ligation into pTripZ-mycBirA^∗^ [57]. GST-Nesprin-1α-326-634 comprising amino acids 326-634 of Nesprin-1α was obtained by PCR from pcDNA3.1-GFP-Nesprin-1α using the following primers: 5′-AAAAAGAATTCGAGCAGCTGATAGAGAAGAGCGAGC-3′ and 5′-AAAAACTCGAGTCAGATGTGAGACTCGATGGTGTGGATGTC-3′ and ligated into pGEX-4T-1 following restriction digest with EcoRI and XhoI. pTripZ-mycBirA^∗^-Nesprin-2β was obtained by PCR of Nesprin-2β coding sequence from HeLa cell cDNA using 5′- GCGCCTCGAGATGTCCATGGAGCGGCGCATG-3′ and 5′-GCGCACGCGTCCTAGGTCATGTGGGGGGTGGCCCATTG-3′, restriction digest with XhoI and MluI and ligation into pTripZ-mycBirA^∗^. Site-directed mutagenesis of Nesprin-1α to obtain Nesprin-1α (WD/AA) was performed using the megaprimer method – the primers 5′-AAGCTCGAGATGGTGGTGGCAGAGGACTTGC-3′ and 5′-TCGTAATCGTGGGCCGCTTCCAGGGGGATGG-3′ were used to generate a forward megaprimer, which was used with reverse primer 5′-AGGCCACGCGTCCTAGGTCAGAGTGGAGGAGG-3′ to PCR amplify mutant Nesprin-1α. Nesprin-1α (WD/AA) was then cloned into pTripZ-mycBirA^∗^ by restriction digestion with XhoI and MluI and ligation. For CRISPR-mediated gene editing, oligos encoding single guide RNA sequences targeting Nesprin-1 (N terminus: ACATCACCAATGTGATGCAG, C terminus: CCGTTGGTATATCTGAGCAT) were annealed by heating to 95°C then cooled gradually to room temperature, then ligated into BbsI-digested pX330-U6-Chimeric BB-CBh-hSpCas9 (a gift from Feng Zhang, Addgene plasmid Cat#42230) [55].

#### Co-transfection of plasmids

For co-transfection of dsRed-PACT and GFP-Nesprin-1α or GFP, respectively, 8-well μ-slides were coated with sterile-filtered 0.1% gelatin/ddH_2_O for 20 min at room temperature. One day prior to transfection, 9.000 C2C12 cells were seeded per well in 200 μL growth medium. When cells reached approximately 80% confluency, cell transfection was carried out using jetPrime. In brief, a total amount of 1 μg DNA was diluted in 50 μL jetPrime buffer and mixed by vortexing vigorously for 10 s. 2 μL jetPrime reagent were added, the mixture was vortexed again for 10 s and shortly spun down. After incubation for 10 min at room temperature, the transfection mix was added drop wise onto the cells which were kept in 200 μL freshly added growth medium containing serum and antibiotics. The following day, the growth medium was replaced to circumvent toxic side effects of the transfection reagent. The cells were kept for 36 hr in growth medium to ensure proper expression, fixed in 4% PFA and stained for Myogenin and DAPI.

#### Nesprin-1 monoclonal antibody production

Expression of GST-Nesprin-1α-326-634 was induced in mid-log phase BL21 (DE3) *E. coli* using 0.1 mM IPTG at 30°C for 2 hr. Pelleted bacteria were resuspended in 4°C phosphate buffered saline with 0.1 mg/ml lysozyme then lysed by several freeze-thaw cycles using liquid nitrogen, followed by sonication to shear DNA. Lysates were cleared by centrifugation at 35000 g for 30 min and filtering the supernatant through a 0.45 μm filter. GST-Nesprin-1α-326-634 was purified from cleared lysate by incubation on Glutathione Sepharose 4B beads for 2 hr at room temperature, washed with 3 × 15 column volumes phosphate buffered saline, and eluted in 10 mM reduced glutathione in 50 mM Tris, pH 8. For monoclonal antibody production mice were immunized intraperitoneally with 50 μg of fusion protein emulsified with Freund’s complete adjuvant. After three weeks a 50 μg boost was administered by the same route in incomplete adjuvant. After additional three weeks, a further 50 μg was administered in PBS. Three days later, the spleen was harvested, minced finely, and passed through a 100 μm cell strainer. SP2/0 myeloma cells were maintained in growth medium (Advance RPMI 1640, 2 mM L-glutamine, 50 μM beta-mercaptoethanol, 10% heat inactivated fetal bovine serum, 100 U/ml penicillin; 100 μg/ml streptomycin). Spleen cells and SP2/0 myeloma cells were washed in GKN saline solution (8 g/L NaCl, 0.4 g/L KCl, 3.56 g/L Na_2_HPO_4_.12H_2_O, 0.78 g/L NaH_2_PO_4_.2H_2_O, 2 g/L Glucose). Spleen cells were fused with 1-2.5 × 10^7^ myeloma cells by gradual addition to cell pellet at 37°C of 1 mL 50% (w/v) polyethylene glycol (Mw ∼1500) over 1 min, incubation for 1 min, addition over 1 min each of 1 mL, 2 mL, 8 mL, then 30 mL GKN saline solution (modified from [58]). Cells were incubated for a further 5 min before pelleting at 300 g for 5 min. Fused cells were distributed into 20 96-well plates in hybridoma medium (Advanced RPMI 1640, 2 mM L-glutamine, 50 μM beta-mercaptoethanol, 15% fetal bovine serum, 20% SP2-conditioned growth medium, 100 U/ml penicillin; 100 μg/ml streptomycin), which is changed to HAT medium (hybridoma medium with 2x HAT supplement) the following day. After 10 days, culture supernatants were screened by immunofluorescence microscopy on NRK cells grown in 96-well plates with optical plastic bottoms. Positive hybridoma cultures were expanded in 24-well plates and single cell cloned using a flow cytometer. Spent culture medium containing antibody from clone 9F10 was employed for all further experiments.

#### BioID proteomics

C2C12 cells were transduced with lentivirus expressing myc-BirA^∗^-Nesprin-1α and stable integration of the transgene was selected for using 0.67 μg/ml puromycin. For each BioID sample, stably transduced cells were expanded in 5-layer flasks and either harvested for myoblast samples, or switched to differentiation medium for 4 days before harvesting for myotube samples. Prior to harvesting, cells were incubated with 50 μM biotin and 1 μg/ml doxycycline for 1-2 days. For control samples, cells were incubated without biotin and doxycycline, or with biotin only. Affinity purification of biotinylated proteins was performed as previously described [13, 57], by lysing and sonicating cells in lysis buffer (50 mM Tris, pH 7.4, 500 mM NaCl, 0.4% SDS, 5 mM EDTA, 1 mM DTT, and 1x complete protease inhibitor). Following addition of Triton X-100 to 2% and further sonication, an equal volume of 50 mM Tris (pH 7.4) was added before additional sonication. Cell lysates were subjected to centrifugation at 16000 relative centrifugal force and supernatants were incubated on MyOne Steptavadin C1 Dynabeads to capture biotinylated proteins. Beads were washed sequentially with wash buffer 1 (2% SDS), wash buffer 2 (0.1% deoxycholate, 1% Triton X-100, 500 mM NaCl, 1 mM EDTA, and 50 mM HEPES, pH 7.5), wash buffer 3 (250 mM LiCl, 0.5% NP-40, 0.5% deoxycholate, 1 mM EDTA, and 10 mM Tris, pH 8.1) and twice with wash buffer 4 (50 mM Tris, pH 7.4, and 50 mM NaCl).

#### Mass spectrometry

Mass spectrometry samples were in-solution digested. After streptavidin affinity purification, beads were denatured using 50% 2,2,2-trifluoroethanol (TFE) in 100 mM triethylammonium bicarbonate (TEAB). Proteins were reduced in 25 mM Tris(2-carboxyethyl)phosphine and alkylated with 55 mM 2-chloroacetamide. Prior digestion sample was further diluted with 100 mM TEAB to achieve TFE final concentration below 5%. Digestion with Lys-C for 4 hr (1:100 enzyme/protein ratio) and Trypsin for 18 hr (1:100) was performed. Following acidification with 1% trifluoroacetic acid, samples were desalted with Sep-Pak C-18 cartridges. Samples were labeled with a TMT (tandem mass tag) isobaric labeling kit in 100 mM TEAB with 10 μL label for 25 μg peptides as suggested in the manufacturer’s instructions. Specifically, each sample was re-suspended in 25 μL TEAB; to tag the samples for multiplex measurement, 10 μL of specific TMT label was added to each sample separately (at 1 Da mass spacing: 126, 127, 128 etc.). Reaction was incubated at room temperature for 1 hr and quenched with 30 μL of 10 mM ammonia formate. Following quenching, samples were mixed and fractionated. Samples were desalted on C18 Basic Resins 10 μm and eluted using a step gradient (4 fractions) containing 17.5%; 25%; 30%; 50% acetonitrile further optimized to (3 fractions) 15%, 22%, 50% acetonitrile in 7.5% ammonium hydroxide. Each fraction was vacuum centrifuged and submitted for analysis. An Orbitrap Fusion mass spectrometer coupled to nanoUPLC Easy LC 1000 system was used for analysis. Samples were injected and separated in EasySpray column 50 cm x 75 μm (C18, 1.8 μm) in a 120 min gradient (Solvent A: 0.1% formic acid; Solvent B: 0.1% formic acid / 99.9% acetonitrile) in data dependent mode using Orbitrap analyzer (speed mode −3 s cycle) with ion targets and resolution (OT-MS 4xE5, resolution 60K, OT-MS/MS 1E5, resolution 15k). Peak lists were generated with Proteome Discoverer 2.1 software and searches were done with Mascot 2.5.1 against concatenated forward/decoy Uniprot/Swiss-Prot with BirA sequences/contaminants database with the following parameters: precursor mass tolerance (MS) 30 ppm, OT-MS/MS 0.06 Da, 3 missed cleavages; static modifications: Carboamidomethyl (C); variable modifications: Oxidation (M), Deamidated (NQ), Acetyl N-terminal protein Phospho (STY), Biotin(K). Forward/decoy searches were used for false discovery rate estimation for PSM and peptides matching high confidence (FDR 1%) and medium confidence (FDR 5%).

#### Mass spectrometry data processing

Following protein identification, extracted TMT reporter abundances were used for protein quantitation. For protein normalization, the bait (BirA-Nesprin-1α) expression was assessed in both myoblasts and myotubes. Using BirA^∗^ specific peptides, correcting factors were derived and used for normalization of specific Nesprin-1α interacting proteins. Proteins identified in at least two biological replicates were used for further analysis.

#### MT re-growth assay

48 hr differentiated C2C12 or human immortalized cells were treated with 5 μg/ml nocodazole for 2 hr in the corresponding differentiation medium. After a quick wash, cells were let to recover in fresh differentiation medium for 4 min (human immortalized cells) or 5 min (C2C12 cells) at 37°C/5% CO_2_. Cells were then pre-extracted for 30 s with 1% Triton X-100 in PHEM buffer (60 mM PIPES, 25 mM HEPES, 10 mM EGTA, 2 mM MgCl_2_, pH 6.9), fixed with 4% paraformaldehyde (PFA) and stained as indicated below.

#### Immunofluorescence

Cells were fixed either in methanol for 20 min at −20°C or in 4% PFA for 15 min at room temperature (RT) and then permeabilized with 0.5% Triton X-100/PBS for 5 min at RT. For detection of Klc1/2 at the NE, cells were pre-extracted for 20 s prior to fixation. After three washes in PBS, cells were blocked in 3% bovine serum albumin or 10% goat serum in PBS for 30 min at RT. Primary antibodies were incubated in blocking buffer for 1-2 hr at RT or at 4°C overnight. Following primary antibody incubation, cells were washed with PBS, incubated with fluorophore-conjugated secondary antibodies and DAPI in blocking buffer for 1 hr at RT, washed with PBS, and mounted in Fluoromount-G or Prolong Diamond or 1% DABCO (1,4-Diazabicyclo[2.2.2]octane) in 10% PBS / 90% glycerol.

#### Imaging

Wide-field epi-fluorescence imaging was performed on an inverted Nikon Ti microscope equipped with a XY-motorized stage using 20x 0.45 NA S Plan Fluor air or 40x 1.3 NA Plan Fluor oil objectives with a 1.5x magnifier. Digital images were acquired with a CoolSNAP HQ2 camera using Metamorph Software and processed in Fiji. For analyses of C2C12 cells after MT re-growth assay, a maximum projection of four 0.25 μm z sections was directly created during imaging using Metamorph. Images were adjusted using the unsharp mask plugin of Fiji. Wide-field epi-fluorescence images were also acquired on a DeltaVision CORE equipped with a xenon light source and bandpass filters with a Plan Apochromat 40 × /1.35 NA oil-immersion objective lens and a CCD camera (no binning; CoolSNAP HQ). For deconvolved images, z-spacing was fixed at 0.2 μm on the DeltaVision CORE and deconvolution then completed using the SoftWorX program. Live-cell imaging of differentiating human immortalized cells was performed for 63 hr in an environmental chamber at 37°C and 5% CO_2_ using a 10x 0.3 NA PL Fluo objective. Confocal images of human immortalized cells after MT re-growth assay and staining were acquired on a Leica SPE confocal microscope using the 63x 1.3 NA Apo objective or on a Nikon Spinning disk confocal with EMCCD camera using a 60x (oil) 1.4 NA objective. Confocal images of C2C12 cells were also obtained on a Zeiss LSM510 confocal microscope using a 40x 1.3 NA objective.

#### SD-*d*STORM

Wild-type C2C12 cells were differentiated for 48 hr, fixed and stained for Pericentrin and Nesprin-1 (MANNES1E antibody) as described above. Alexa Fluor 647 and CF 680 labeled secondary antibodies were used. This dye pair has previously been shown to be suitable for spectral demixing (SD)-*d*STORM [26]. For SD-*d*STORM, coverslips were mounted in imaging buffer (50 mM Tris/HCl, 10 mM NaCl, pH 8) containing 10 mM β-mercaptoethylamine (MEA), 0.5 mg/ml glucose oxidase, 40 μg/ml catalase, and 10% (w/v) glucose on slides with molds. Images were acquired on a custom-build SD-*d*STORM system using a 700-DCXXR dichroic mirror and a F76-635 emission bandpass filter and reconstructed as described previously [25, 26].

#### Structured illumination microscopy (SIM)

Wild-type C2C12 cells were differentiated for 48 hr, subjected to MT re-growth assay, fixed and stained as described above. Coverslips were mounted using Vectashield and sealed with nail polish. 3D-SIM images were generated using the 488 nm, 568 nm, and 643 nm laser lines and standard filter sets on the OMX V4 Blaze. Precise registration (within ∼40 nm) of the three-color channels was verified using TetraSpeck multicolor beads mounted on coverslips.

#### SDS-PAGE and Western Blot analysis

C2C12 cells were lysed in 1% SDS/PBS and homogenized by full-speed centrifugation for one minute using a QIAshredder. Total protein concentration was determined using the Pierce bicinchoninic acid (BCA) protein assay kit. Equal protein amounts were mixed with 4x Laemmli sample buffer, heated for 5 min at 95°C, loaded on 4%–15% Mini-PROTEAN TGX protein gels and transferred onto a nitrocellulose membrane using the Trans-Blot Turbo Transfer system. For analysis of high molecular weight proteins, samples were loaded on 4%–12% NuPAGE Novex Bis-Tris gels and transferred over night at 30 V in 1x Tris/Glycine buffer containing 10% ethanol and 0.025% SDS. Membranes were blocked in 5% milk/0.05% Tween-TBS and incubated with primary antibodies over night at 4°C. After incubation with HRP-conjugated secondary antibodies for 1 hr at room temperature, proteins were detected by chemiluminescence using SuperSignal West Pico Chemiluminescence or Luminata Forte Western HRP Substrate and Amersham Hyperfilms. For western blots scanned on an Odyssey imaging system, IRDye 800 or IRDye 680 conjugated secondary antibody or streptavidin was used.

#### Computer simulation

Computer simulations were performed using Cytosim [50]. In brief, overdamped Langevin equations are used to describe the motion of elastic fibers and nuclei in a viscous fluid in the presence of Brownian motion. All stochastic events (motor binding, MT catastrophes, and MT nucleation) are generated as first-order random events. The simulation parameters are summarized in Table S1. Myotubes have elliptical shapes, 95 × 14 μm for five nuclei, 114 × 14 μm for six nuclei, 133 × 14 μm for seven nuclei, 152 × 14 μm for eight nuclei and 171 × 14 μm for nine nuclei, with the cytoplasm having homogeneous constant viscosity, as shown experimentally (Figure S4B). The elliptical boundary confines MTs, centrosomes, and nuclei. A soft excluded volume interaction applies to centrosomes, MTs, and nuclei, preventing these objects from overlapping, but MTs only interact with each other via molecular motors (MT-MT steric interactions are not considered). Nuclei nucleate MTs, which undergo dynamic instability with no rescues. Moreover, the growth rate is reduced by force with a sensitivity of 1.5 pN and depends on the availability of tubulin monomers, for which a fixed pool is prescribed. The catastrophe rate depends on growth rate as observed *in vitro* [59]. MTs are nucleated horizontally, leading to their organization parallel to the long axis of the myotube, as shown by the EB1 comets angle analysis (Figures S4C and S4D). MTs interact with nuclei via the dynein and kinesin motor proteins located at the NE. A given density of motor proteins is evenly distributed on nuclear surfaces. All motor proteins exert Hookean forces and move along bound MTs with a linear force-velocity relationship. The maximum number of MTs nucleated by one nucleus has been estimated by counting the number of EB1 comets near myotube nuclei.

#### Experimental Design

All experiments were performed at least twice, typically 3-4 times. To ensure randomization, for quantification of cellular phenotypes, all cells meeting particular criteria (e.g., for nuclear spreading quantification, only myotubes with 3 or more nuclei were considered) in a field of view were counted. While blinding was not used in this study, where possible, automated image analysis was used. Sample-size estimation and statistical method of computation was not performed as it is relatively easy to obtain large numbers (N > 100) in cell microscopy-based experiments. Inclusion and exclusion criteria of any data or subjects is described in the methods and include quantification of only Myogenin or myosin heavy chain positive nuclei to identify differentiated myotubes, and nuclear spreading analysis on myotubes with at least 3 nuclei.

### Quantification and Statistical Analysis

#### Quantification centrosomal proteins at the NE

We counted the number of nuclei with more than 50% of Pericentrin or Akap450, respectively, at the NE in Myogenin-(MYOG)- or myosin heavy chain-(MHC)-positive cells.

#### Quantification MT re-growth

MT re-growth was quantified by counting the number of Myogenin-positive nuclei that showed at least 50% of MT seeds at the NE.

#### Quantification dsRed-PACT at the NE

We counted the number of Myogenin-negative cells that showed NE distribution of the dsRed-PACT construct in cells co-expressing GFP or GFP-Nesprin-1α, respectively.

#### Statistical Analysis

Statistical tests were performed using GraphPad Prism and are described in each figure legend. The exact value of n is indicated within the respective figure. Statistical significance is represented as follows: ^∗∗∗^ p < 0.001; ^∗∗^ p < 0.01, ^∗^p < 0.05; n.s., not statistically significant.

### Data and Software Availability

Mass spectrometry data is provided in supplemental material as Data S1.

## Author Contributions

P.G., Y.L.L., B.B., B.C., and E.R.G. conceived and designed experiments; P.G., Y.L.L., R.M.S., V.K., and R.P. performed experiments and analyzed data; B.C. performed computational analysis; A.C., K.M., F.N., and J.S. provided resources; P.G. wrote the manuscript; P.G., Y.L.L., B.B., B.C., and E.R.G. revised the manuscript; and S.S., J.S., B.B., B.C., and E.R.G. supervised the project.

## Supplemental Information

Document S1. Figures S1–S4 and Table S1

Table S2. Additional Oligonucleotides

Movie S1. Nuclei Are Mispositioned in *SYNE1* (23560 G>T) Patient Myotubes, Related to Figure 4 Nuclear spreading movement in human control myotubes or in *SYNE1* (23560 G>T) patient myotubes monitored by live-cell imaging.

Movie S2. Computer Simulations Reveal a Role for MT Nucleation from the NE in Nuclear Positioning, Related to Figure 4 Computer simulation of nuclear movements in myotubes with active MT nucleation from the NE and Kif5b motor proteins at the nucleus (top panel), in myotubes without active MT nucleation from the NE but with Kif5b motor proteins retained at the nucleus (middle panel) or in myotubes without active MT nucleation from the NE and without Kif5b motor proteins at the nucleus.

Data S1. Proteins Identified in at Least 2 out of 3 BioID-Nesprin-1α Experiments, Related to Figure 1 Average values are shown for the ratio of BioID affinity purifications performed on myoblasts or myotubes with biotin and doxycycline compared to with biotin and without doxycycline following normalization to the amount of bait. Proteins are ranked according to the ratio of the myotube to myoblast normalized average values.

Document S2. Article plus Supplemental Information
