# Supplementary material for: Nesprin-1α-Dependent Microtubule Nucleation from the Nuclear Envelope via Akap450 Is Necessary for Nuclear Positioning in Muscle Cells
Source: Curr Biol. 2017 Oct 9;27(19):2999–3009.e9. doi: 10.1016/j.cub.2017.08.031 (PMC5640514; doi:10.1016/j.cub.2017.08.031)
Supplement: Document S1. Figures S1–S4 and Table S1 [file mmc1.pdf]

Current Biology, Volume *27*

Supplemental Information

Nesprin-1α-Dependent Microtubule Nucleation from the Nuclear Envelope via Akap450 Is Necessary for Nuclear Positioning in Muscle Cells

Petra Gimpel, Yin Loon Lee, Radoslaw M. Sobota, Alessandra Calvi, Victoria Koullourou, Rutti Patel, Kamel Mamchaoui, François Nédélec, Sue Shackleton, Jan Schmoranzer, Brian Burke, Bruno Cadot, and Edgar R. Gomes

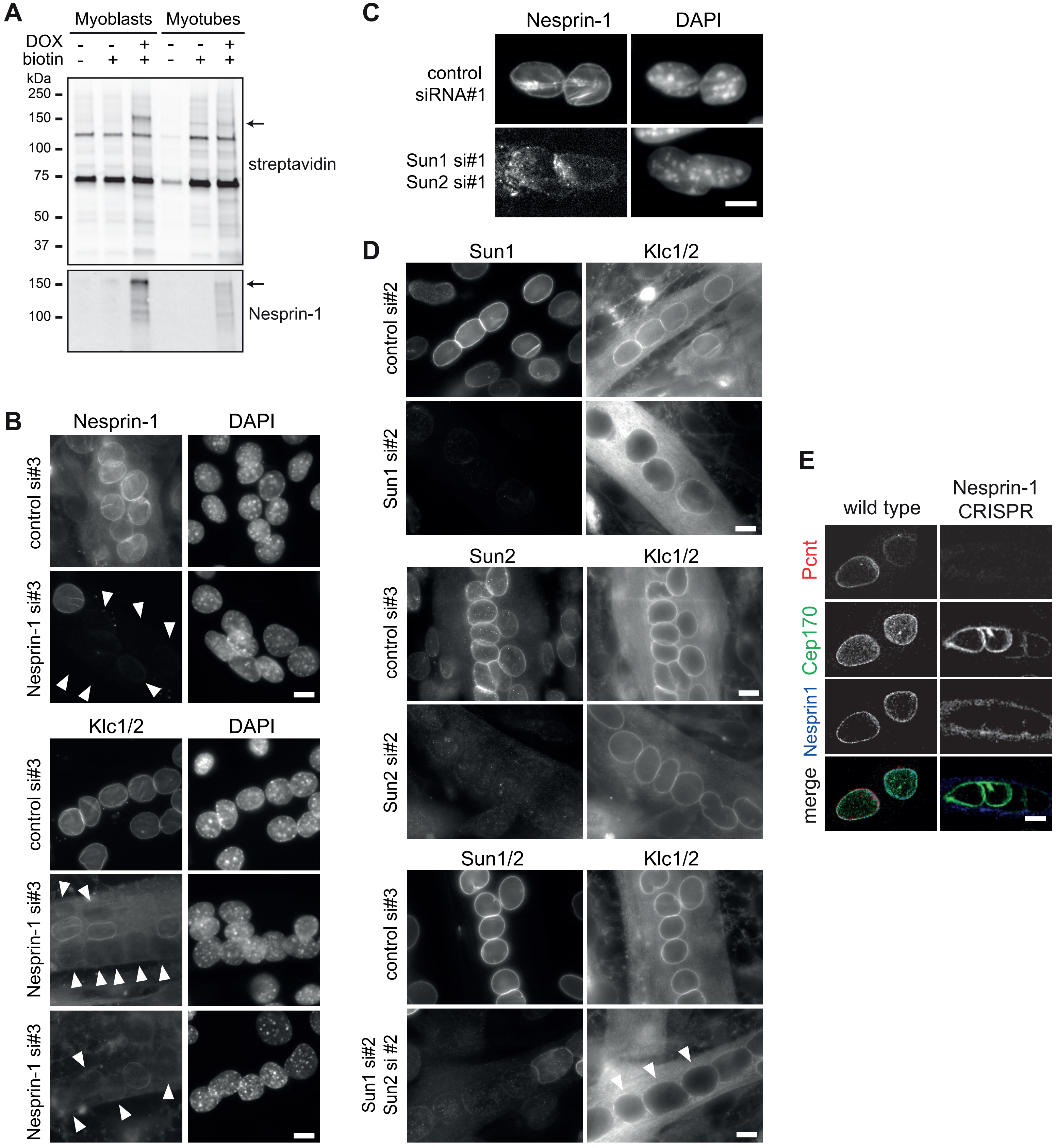

Figure S1. BioID of Nesprin-1α identifies known and novel proximal proteins. Related to Figure 1. (A) C2C12 myoblasts or myotubes stably expressing myc-BirA*-Nesprin-1α were treated with or without doxycycline and biotin, harvested and subjected to affinity purification using streptavidin-beads. Expression of myc-BirA*-Nesprin-1α after doxycycline addition was confirmed by Western Blot using anti-Nesprin-1 (clone 9F10) antibody. Biotinylated proteins were detected using fluorescent conjugates of streptavidin. Arrows point to BioID-Nesprin-1α. **(B)** C2C12 myotubes were transfected with the indicated siRNAs and stained for Nesprin-1 (MANNES1A) or Klc1/2 and nuclei (DAPI). Arrowheads indicate myonuclei with loss of Klc1/2 localisation to the NE following Nesprin-1 knockdown. Scale bar, 10 µm. **(C)** 48 h differentiated C2C12 cells were transfected with the indicated siRNAs and stained for Nesprin-1 (MANNES1E) and nuclei (DAPI). Scale bar, 10 µm. **(D)** C2C12 myotubes were transfected with the indicated siRNAs and stained for Klc1/2 and nuclei (DAPI). Arrowheads indicate myonuclei with loss of Klc1/2 localisation to the NE following double Sun1/Sun2 knockdown. Scale bar, 10 µm. **(E)** Wild type or Nesprin-1 CRISPR mutant C2C12 myotubes were stained for Pericentrin (Pcnt, red), Cep170 (green), or Nesprin-1 (blue, 9F10). Scale bar, 10 µm.

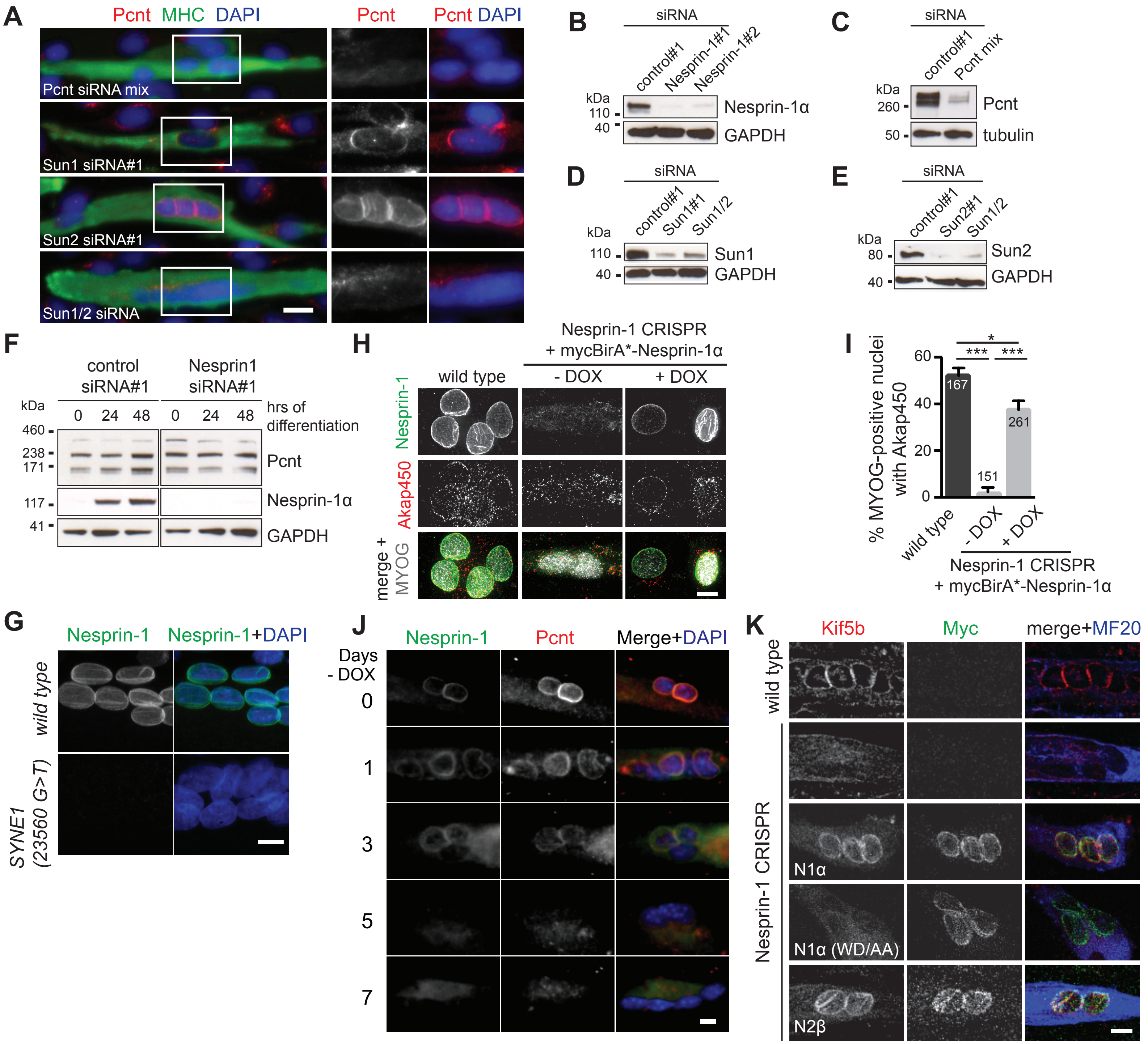

Figure S2. Nesprin-1α-containing LINC complex recruits centrosomal proteins to the myotube NE. Related to Figure 2. **(A)** 48 h differentiated C2C12 cells were transfected with the indicated siRNAs and stained for Pericentrin (Pcnt, red), nuclei (DAPI, blue) and myosin heavy chain (MHC, green). Scale bar, 20 µm. **(B, C, D, E)** Western Blot of 48 h differentiated C2C12 cells transfected with the indicated siRNAs, stained for Nesprin-1α (MANNES1E) (B), Pericentrin (Pcnt) (C), Sun1 (D) or Sun2 (E), respectively, and for GAPDH or tubulin (YL 1/2) as a loading control. **(F)** C2C12 cells were treated with non-targeting control siRNA#1 or Nesprin-1 siRNA#1, differentiated for the indicated time points (hours of differentiation), subjected to SDS-PAGE and Western Blot analysis using anti-Pericentrin (Pcnt), anti-Nesprin-1α (MANNES1E) and anti-GAPDH antibodies. **(G)** Differentiated human immortalized myotubes from a healthy control (wild type) or from a patient carrying a nonsense mutation within the *SYNE1 (23560 G>T)* gene immunostained for Nesprin-1α/Nesprin-1 (MANNES1E, green) and nuclei (DAPI, blue). Scale bar, 10 µm. **(H)** C2C12 wild type or Nesprin-1 CRISPR mutant cells transduced with mycBirA*-Nesprin-1α without and with 1 µg/ml doxycycline (-/+DOX) were differentiated for 48 h, fixed and stained for Nesprin-1 (green, clone 9F10), Akap450 (red), and Myogenin (MYOG, grey) Scale bar, 10 µm. **(I)** Quantification of Akap450 recruitment to the NE in Myogenin (MYOG)-positive nuclei as described in (H). Error bars ± SEM. n represents total number of nuclei from three independent experiments. ***$p<0.001$, *$p<0.05$, one-way ANOVA with Tukey's multiple comparisons test. **(J)** Nesprin-1 CRISPR mutant C2C12 myotubes transduced with mycBirA*-Nesprin-1α were incubated with differentiation media containing doxycycline (DOX) for at least 24 hours and then switched to differentiation media lacking (-) doxycycline (DOX) for 0-7 days as indicated. Cells were then fixed and stained for Nesprin-1 (green, 9F10), Pericentrin (Pcnt, red) and nuclei (DAPI, blue). Scale bar, 10 µm. **(K)** C2C12 wild type, untransduced Nesprin-1 CRISPR mutant cells or CRISPR mutant cells transduced with mycBirA*-Nesprin-1α (N1α), mycBirA*-Nesprin-1α with the LEWD motif mutated to LEAA (N1α (WD/AA)), or mycBirA*-Nesprin-2β (N2β) were incubated with doxycycline and differentiated for 48 h, fixed and stained for Kif5b (red), Myc (green) and myosin heavy chain (clone MF20, blue). Scale bar, 10 µm.

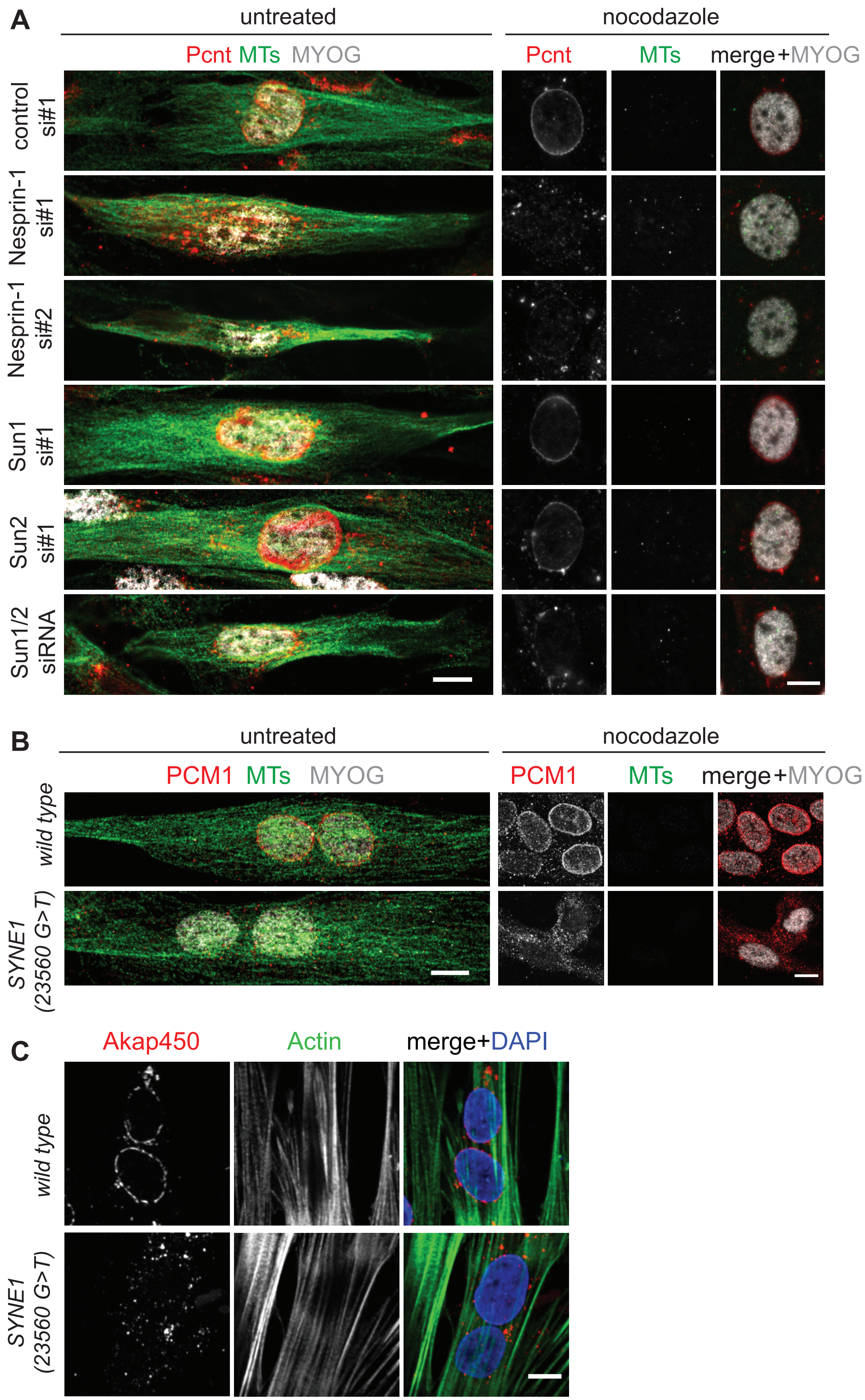

Figure S3. Disruption of LINC complex does not affect overall microtubule or actin organization. Related to Figure 3. (A) 48 h differentiated C2C12 cells, treated with non-targeting control (NC) siRNA#1, two different siRNAs to Nesprin-1 (Nesprin-1 #1 or Nesprin-1 #2), Sun1, Sun2 or both Sun1 and Sun2 (Sun1/2) siRNAs, were incubated with or without (untreated) nocodazole and immunostained for Pericentrin (Pcnt, red), microtubules (MTs, green) and Myogenin (MYOG, grey). Scale bar, 10 µm. **(B)** Human immortalized myotubes from a healthy control (wild type) or from a patient carrying a nonsense mutation within the *SYNE1 (23560 G>T)* gene were treated with or without (untreated) nocodazole and immunostained for PCM1 (red), microtubules (MTs, green) and nuclei (DAPI, blue). Scale bar, 10 µm. **(C)** Human immortalized myotubes from a healthy control (wild type) or from a patient carrying a nonsense mutation within the *SYNE1 (23560 G>T)* gene were stained for Akap450 (red), actin (green, phalloidin) and nuclei (DAPI, blue). Scale bar, 10 µm.

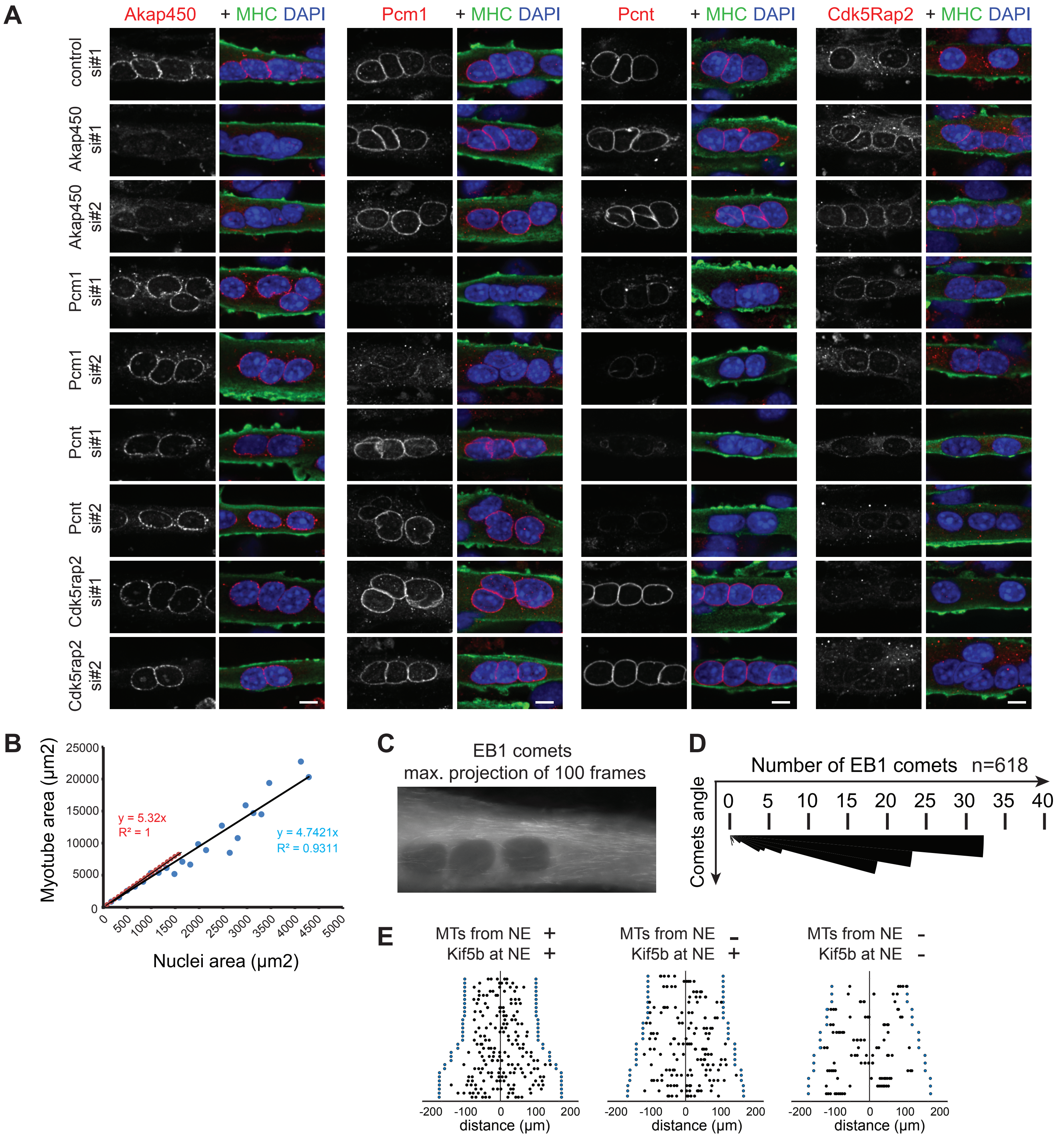

Figure S4. Computer simulations reveal role for Akap450-mediated MT nucleation in nuclear positioning independent of other centrosomal proteins. Related to Figure 4. (A) C2C12 cells were transfected with the indicated siRNAs, differentiated into myotubes for 48 hours and stained for Akap450, Pcm1, Pericentrin (Pcnt) or Cdk5Rap2 (red), myosin heavy chain (MHC, green) and nuclei (DAPI, blue). Scale bar, 10 μm. **(B)** The myotube area was plotted over the number of nuclei for C2C12 myotubes (blue dots) or for simulated myotubes generated using Cytosim (red dots). **(C)** EB1-GFP-expressing myotubes were imaged using stream acquisition (250 ms/frame). We used a maximum projection of 100 frames to measure the angle of each comet compared to the long axis of the myotube. **(D)** All comet angles were then normalized over a 90° quadrant and distributed in 5° steps, thus revealing a preferential orientation of EB1 comets towards the long axis of the myotube. **(E)** Two-dimensional representations of nuclear distributions in myotubes after computer simulations. We compared nuclear positioning in myotubes with MT nucleation activity and Kif5b motor proteins at the NE (control; MTs from NE+, Kif5b at NE+), without MT nucleation activity but Kif5b motor proteins at the NE (MTs from NE-; Kif5b at NE+) or without both MT nucleation activity and Kif5b motor proteins at the NE (MTs from NE-; Kif5b at NE-). Thereby, each myotube is represented on a single line with blue dots representing the myotube edges and black dots the nuclei.

Name	Value	Note
**Global**		
Time step	0.05s	Computational parameter
Viscosity	0.1pN s/mm²	Estimate viscosity of the cytoplasm, [S1]
kT	0.0042 pN mm	Thermal energy at 25°C
Cell geometry	R=7um L=190 mm / 5 nuclei	Ellipse
	L=228 mm / 6 nuclei	
	L=266mm / 7 nuclei	
	L=304mm / 8 nucleis	
	L=342mm / 9 nuclei	
**Microtubules**		
Rigidity	20 pN mm²	Flexural rigidity [S2-S4]
Segmentation	4 mm	computational parameter
Dynamics	growing speed= 0.4 mm/s	measures (growing speed only)
	Shrinking speed= 0.8 mm/s	[S5]
	hydrolysis rate= 0.5 mm/s	[S5]
Growing forces	fg=1.5pN	growing velocity is slowed down by antagonostic force on plus end.
	Stiffness = 500 pN/ mm	
Total polymer	7000 per nucleus	to limit the length of MTs
**Kinesin (Kif5b)**		
Binding	Range= 0.05 mm	Maximal distance to which a binder can bind a filament
	Rate = 5 s^{-1}	Rate at which possible binding can occur
Unbinding	Force= 5 pN	Unbinding increases with load exponentially
	Rate= 0.1 s^{-1}	[S6]
Stiffness	200pN/mm	
Motility	Max speed vm= 0.8 mm/s	[S6]
	stall force fs=5 pN	
**Crossslider kif5b/Map7 like**		
Diffusion	20 mm²/s	
Stiffness	500pN	
Activity	Slide	Map7 moves in the direction of the applied force, with the specified mobility
Binding	Range= 0.1mm	Maximal distance to which a binder can bind a filament
	Rate = 10 s^{-1}	Rate at which possible binding can occur
Unbinding	Force = 1 pN	Load force needed to unbind
	Rate = 10 s^{-1}	Rate at which possible unbinding can occur
Motility	0.1 $mm.s^{-1}.pN^{-1}$	
Quantity	200 per nucleus	
**Map4**		
Binding	Range= 0.1 mm	Maximal distance to which a binder can bind a filament
	Rate = 2 s^{-1}	Rate at which possible binding can occur
Unbinding	Force= 3 pN	Load force needed to unbind
	Rate= 1 s^{-1}	Rate at which possible unbinding can occur
**Crosslinker Map4/Map4**		
Diffusion	20 mm²/s	
Stiffness	k=200pN	
Activity	Bridge	[S7]
Specificity	Antiparallel	[S7]
Quantity	400 per nucleus	
**Dynein**		
Binding	Range= 0.05 mm	[S8], [S9]
	Rate = 5 s^{-1}	
Unbinding	Force= 3 pN	[S10]
	Rate= 0.1 s^{-1}	motors unbinding rate is deduced from measurements of dynein processivity ~ 1-2 µm (reviewed in [S9]) and dynein velocity ~ 1.5 µm s-1
Stiffness	k=200 pN/mm	
Motility	Max speed vm= 1 mm/s	[S11], [S12], [S3]; reviewed in [S9]
	stall force fs=5 pN	Measurements ~1-7 ([S11], [S13]; reviewed in [S12] and [S9]
**Nuclei**		
Quantity	5 to 9	Density is 1 nuclei/ 700um² of myotube (measured)
Radius	5 mm	
Nucleator (Gamma-tubulin)	Quantity = 58 / nucleus	The nucleators nucleate independently.
	Nucleation rate = 0.02 s^{-1}	50 possible new MTs per second and per nucleator
	Unbinding rate = 0.1s^{-1}	
	Unbinding force= 3 pN	
	Stiffness = 1000pN/mm	
Kif5b	Quantity = 30 / nucleus	Fixed at the NE, stiffness = 200pN/um
Dynein	Quantity = 18 / nucleus	Fixed at the NE, stiffness = 200pN/um
**Centrioles (when no Nesprin-1)**		
Quantity	5 per nucleus	
Radius	0.2 mm	
MT nucleation sites	11	

Table S1. Parameters for the Cytosim computer simulations. Related to Figure 4G.

Supplemental References

S1. Daniels, B.R., Masi, B.C., and Wirtz, D. (2006). Probing Single-Cell Micromechanics In Vivo: The Microrheology of C. elegans Developing Embryos. Biophysical Journal *90*, 4712–4719.

S2. Dogterom, M., and Yurke, B. (1997). Measurement of the Force-Velocity Relation for Growing Microtubules. Science *278*, 856–860.

S3. Kimura, A., and Onami, S. (2005). Computer simulations and image processing reveal length-dependent pulling force as the primary mechanism for C. elegans male pronuclear migration. Dev. Cell *8*, 765–775.

S4. Kozlowski, C., Srayko, M., and Nedelec, F. (2007). Cortical microtubule contacts position the spindle in C. elegans embryos. Cell *129*, 499–510.

S5. Brun, L., Rupp, B., Ward, J.J., and Nédélec, F. (2009). A theory of microtubule catastrophes and their regulation. Proc. Natl. Acad. Sci. U.S.A. *106*, 21173–21178.

S6. Andreasson, J.O., Milic, B., Chen, G.-Y., Guydosh, N.R., Hancock, W.O., and Block, S.M. (2015). Examining kinesin processivity within a general gating framework. eLife *4*, e07403.

S7. Mogessie, B., Roth, D., Rahil, Z., and Straube, A. (2015). A novel isoform of MAP4 organises the paraxial microtubule array required for muscle cell differentiation. eLife Sciences *4*, e05697.

S8. De Simone, A., Nédélec, F., and Gönczy, P. (2016). Dynein Transmits Polarized Actomyosin Cortical Flows to Promote Centrosome Separation. Cell Rep *14*, 2250–2262.

S9. Goodman, B.S., Derr, N.D., and Reck-Peterson, S.L. (2012). Engineered, harnessed, and hijacked: synthetic uses for cytoskeletal systems. Trends Cell Biol. *22*, 644–652.

S10. Rupp, B., and Nédélec, F. (2012). Patterns of molecular motors that guide and sort filaments. Lab Chip *12*, 4903–4910.

S11. Athale, C.A., Dinarina, A., Nedelec, F., and Karsenti, E. (2014). Collective behavior of minus-ended motors in mitotic microtubule asters gliding toward DNA. Phys Biol *11*, 16008.

S12. Schief, W.R., and Howard, J. (2001). Conformational changes during kinesin motility. Curr. Opin. Cell Biol. *13*, 19–28.

S13. Goshima, G., Nédélec, F., and Vale, R.D. (2005). Mechanisms for focusing mitotic spindle poles by minus end-directed motor proteins. J. Cell Biol. *171*, 229–240.

S14. Haque, F., Lloyd, D.J., Smallwood, D.T., Dent, C.L., Shanahan, C.M., Fry, A.M., Trembath, R.C., and Shackleton, S. (2006). SUN1 interacts with nuclear lamin A and cytoplasmic nesprins to provide a physical connection between the nuclear lamina and

the cytoskeleton. Mol. Cell. Biol. *26*, 3738–3751.
