# Supplementary material for: Nesprin-1α-Dependent Microtubule Nucleation from the Nuclear Envelope via Akap450 Is Necessary for Nuclear Positioning in Muscle Cells
Source: Curr Biol. 2017 Oct 9;27(19):2999–3009.e9. doi: 10.1016/j.cub.2017.08.031 (PMC5640514; doi:10.1016/j.cub.2017.08.031)
Supplement: Table S2. Additional Oligonucleotides [file mmc2.docx]

| REAGENT or RESOURCE | SOURCE | IDENTIFIER |
| --- | --- | --- |
| Oligonucleotides | | |
| siRNA targeting sequence: mouse Nesprin-1 #3  CAGAGUUGGCCAAGCCCAUAGUCUA | Invitrogen | Cat#10620312-249678B12/ 249678C01 |
| siRNA targeting sequence: mouse Sun1 #2  CCAACUUGGAAGAUGUUCUUAGAAA | [S14] | N/A |
| siRNA targeting sequence: mouse Sun2 #2  CATACCAAGTTGTGGAGCTTCGGAT | [S14] | N/A |
| siRNA targeting sequence: mouse Cdk5rap2 #1  GAGAUCACCUUGAUAGUAAtt | Ambion | Cat#4390771-s103085 |
| siRNA targeting sequence: mouse Cdk5rap2 #2  CAGUGAGGCUAUUAUCACAtt | Ambion | Cat#4390771- s103086 |
| siRNA non-targeting control #1  UUCUCCGAAGCUGUCACGUtt | GeneCust | N/A |
| siRNA non-targeting control #2  CGUUAAUCGCGUAUAAUACGCGUAT | Integrated DNA Technologies | Cat#51-01-14-03 |
| siRNA non-targeting control #3  CGUACGCGGAAUACUUCGATT | GE Dharmacon | Cat#D-001100-01-20 |
| Primer mouse Nesprin-1α forward (pcDNA3.1)  GCGCCTCGAGATGGTGGTGGCAGAGGACTTGC | This paper | N/A |
| Primer mouse Nesprin-1α reverse (pcDNA3.1)  GCGCCTTAAGTCAGAGTGGAGGAGGACCGTT | This paper | N/A |
| Primer mouse Nesprin-1α forward (pTripZ)  AAGCTCGAGATGGTGGTGGCAGAGGACTTGC | This paper | N/A |
| Primer mouse Nesprin-1α reverse (pTripZ) AGGCCACGCGTCCTAGGTCAGAGTGGAGGAGG | This paper | N/A |
| Primer human Nesprin-2β forward (pTripZ)  GCGCCTCGAGATGTCCATGGAGCGGCGCATG | This paper | N/A |
| Primer human Nesprin-2β reverse (pTripZ)  GCGCACGCGTCCTAGGTCATGTGGGGGGTGGCCCATTG | This paper | N/A |
| Primer GST-Nesprin-1α-326-634 forward  AAAAAGAATTCGAGCAGCTGATAGAGAAGAGCGAGC | This paper | N/A |
| Primer GST-Nesprin-1α-326-634 reverse  AAAAACTCGAGTCAGATGTGAGACTCGATGGTGTGGATGTC | This paper | N/A |
| Primer Nesprin-1α (WD/AA) forward  AAGCTCGAGATGGTGGTGGCAGAGGACTTGC | This paper | N/A |
| Primer Nesprin-1α (WD/AA) reverse  TCGTAATCGTGGGCCGCTTCCAGGGGGATGG | This paper | N/A |
| Primer Nesprin-1α (WD/AA) reverse  AGGCCACGCGTCCTAGGTCAGAGTGGAGGAGG | This paper | N/A |
| Forward oligo for CRISPR single guide RNA targeting Nesprin-1, N-terminus  caccgACATCACCAATGTGATGCAG | This paper | N/A |
| Reverse oligo for CRISPR single guide RNA targeting Nesprin-1, N-terminus  aaacCTGCATCACATTGGTGATGTc | This paper | N/A |
| Oligo for CRISPR single guide RNA targeting Nesprin-1, C-terminus  caccgCCGTTGGTATATCTGAGCAT | This paper | N/A |
| Oligo for CRISPR single guide RNA targeting Nesprin-1, C-terminus  aaacATGCTCAGATATACCAACGGc | This paper | N/A |
