# Supplementary figures and images for: Nesprin-1α-Dependent Microtubule Nucleation from the Nuclear Envelope via Akap450 Is Necessary for Nuclear Positioning in Muscle Cells
Source: Curr Biol. 2017 Oct 9;27(19):2999–3009.e9. doi: 10.1016/j.cub.2017.08.031 (PMC5640514; doi:10.1016/j.cub.2017.08.031)

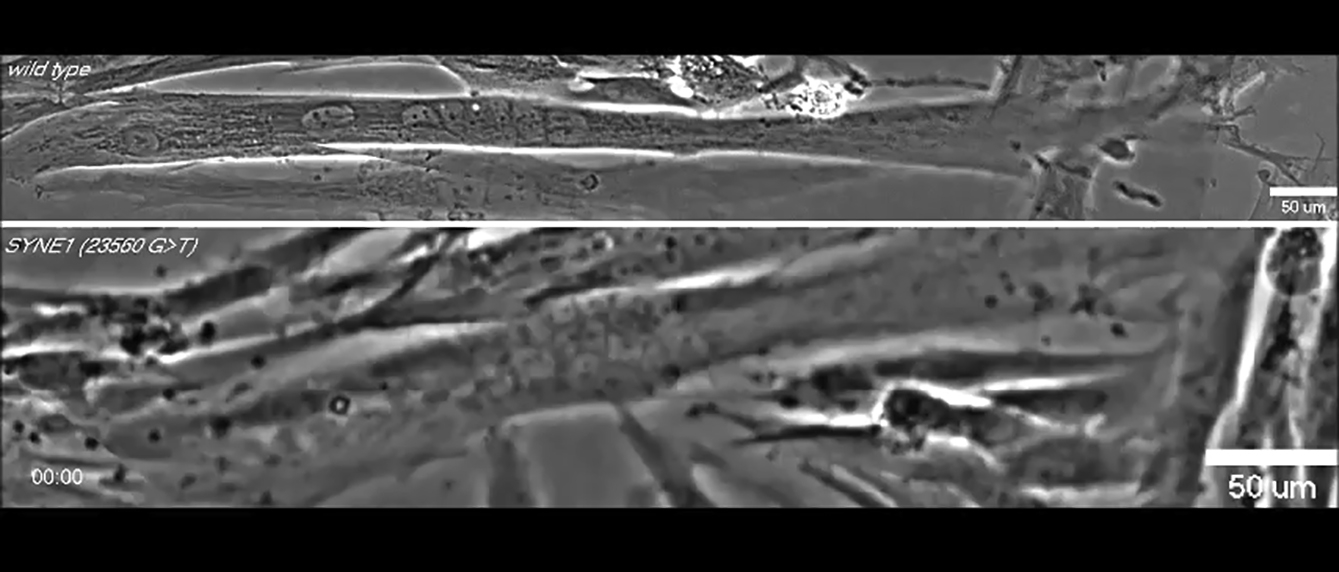

Supplement: Movie S1. Nuclei Are Mispositioned in SYNE1 (23560 G>T) Patient Myotubes, Related to Figure 4 — Nuclear spreading movement in human control myotubes or in SYNE1 (23560 G>T) patient myotubes monitored by live-cell imaging. [file mmc3.jpg]

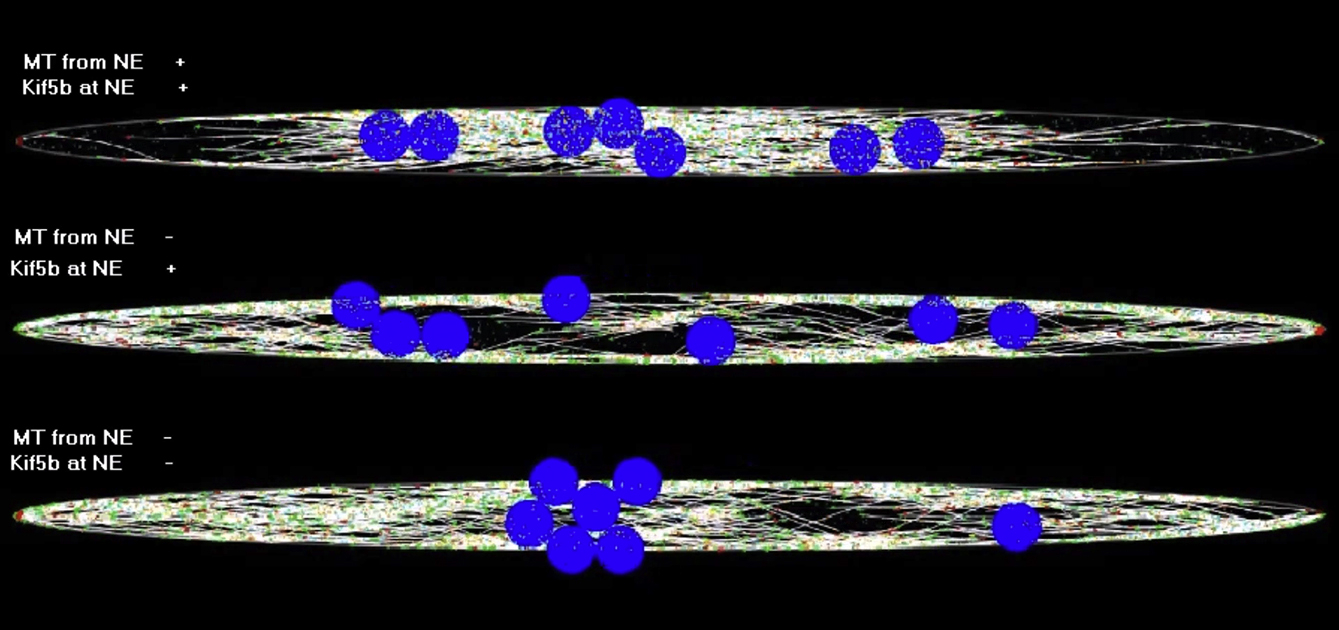

Supplement: Movie S2. Computer Simulations Reveal a Role for MT Nucleation from the NE in Nuclear Positioning, Related to Figure 4 — Computer simulation of nuclear movements in myotubes with active MT nucleation from the NE and Kif5b motor proteins at the nucleus (top panel), in myotubes without active MT nucleation from the NE but with Kif5b motor proteins retained at the nucleus (middle panel) or in myotubes without active MT nucleation from the NE and without Kif5b motor proteins at the nucleus. [file mmc4.jpg]
